# Re-using biological devices: a model-aided analysis of interconnected transcriptional cascades designed from the bottom-up

**DOI:** 10.1186/s13036-017-0090-3

**Published:** 2017-12-14

**Authors:** Lorenzo Pasotti, Massimo Bellato, Michela Casanova, Susanna Zucca, Maria Gabriella Cusella De Angelis, Paolo Magni

**Affiliations:** 1 Laboratory of Bioinformatics, Mathematical Modelling and Synthetic Biology, Department of Electrical, Computer and Biomedical Engineering, University of Pavia, 27100 Pavia, Italy; 2 Centre for Health Technologies, University of Pavia, 27100 Pavia, Italy

**Keywords:** Bottom-up design, Interconnection, Mathematical modelling, Modularity, Cell burden

## Abstract

**Background:**

The study of simplified, ad-hoc constructed model systems can help to elucidate if quantitatively characterized biological parts can be effectively re-used in composite circuits to yield predictable functions. Synthetic systems designed from the bottom-up can enable the building of complex interconnected devices via rational approach, supported by mathematical modelling. However, such process is affected by different, usually non-modelled, unpredictability sources, like cell burden.

**Methods:**

Here, we analyzed a set of synthetic transcriptional cascades in *Escherichia coli*. We aimed to test the predictive power of a simple Hill function activation/repression model (no-burden model, NBM) and of a recently proposed model, including Hill functions and the modulation of proteins expression by cell load (burden model, BM). To test the bottom-up approach, the circuit collection was divided into training and test sets, used to learn individual component functions and test the predicted output of interconnected circuits, respectively.

**Results:**

Among the constructed configurations, two test set circuits showed unexpected logic behaviour. Both NBM and BM were able to predict the quantitative output of interconnected devices with expected behaviour, but only the BM was also able to predict the output of one circuit with unexpected behaviour. Moreover, considering training and test set data together, the BM captures circuits output with higher accuracy than the NBM, which is unable to capture the experimental output exhibited by some of the circuits even qualitatively. Finally, resource usage parameters, estimated via BM, guided the successful construction of new corrected variants of the two circuits showing unexpected behaviour.

**Conclusions:**

Superior descriptive and predictive capabilities were achieved considering resource limitation modelling, but further efforts are needed to improve the accuracy of models for biological engineering.

**Electronic supplementary material:**

The online version of this article (10.1186/s13036-017-0090-3) contains supplementary material, which is available to authorized users.

## Background

Modularity of parts is one of the key features of the engineering world that enables to achieve predictable outcomes upon interconnection of quantitatively characterized components [1]. Even when modularity of components does not persist, i.e., they do not maintain their intrinsic properties when connected with other modules, engineers can still rely on the knowledge of parts interactions to yield predictable systems [1–3]. The predictability of the designed circuits is also a central issue in synthetic biology, since only in a predictable framework biological systems can be constructed from the bottom-up. Mathematical models can support the design process, enabling the rational engineering of complex systems and avoiding trial and error approaches [3–5]. Although standardized approaches for the characterization of parts have been recently proposed, the intrinsic complexity of biological components currently limits the predictability of parts function when they are re-used in different contexts [6, 7]. The major unpredictability sources for biological components are context-dependent and cell-to-cell variability, cross-talk, evolutionary stability, retroactivity and cell burden [2, 6]. Efforts towards the reproducible characterization of parts function include standardized measurement approaches for transcriptional activity [8] and biophysical models for the quantification of ribosome binding sites (RBSs) strength [9, 10] or transcriptional terminators efficiency [11, 12].

The study of simplified model systems can help to elucidate the feasibility boundaries of the bottom-up design approach in biological engineering. Within this framework, different studies used mathematical models to study the superposition of the effects of multiple independent gene expression cassettes [13], context-dependent variability of individual or interconnected devices [14–16], retroactivity effects due to the interconnection of biological modules which share common resources [17] and prediction of quantitative behaviour of logic functions [18–20] or feed-forward circuits [21]. High-throughput studies have also been carried out to evaluate the variation of parts activity in a large number of diverse expression systems, showing the variations expected for promoters, RBSs and genes with different codon composition [22, 23]. Efforts towards the improvement of biological components modularity have recently been carried out by proposing insulated promoters [24, 25], a bicistronic design for gene expression cassettes that makes RBS efficiency more predictable [26], a device for timescale separation to mitigate retroactivity [27] and ribozyme-based insulators at 5′-UTR [28]. Recent works have also proposed methods to guide biological engineers in parts selection, via statistical analyses to evaluate promoter and RBS collections [29], and a computer-aided design tool for the choice of logic devices to construct reliable functions [30]. In the latter study, devices are selected via model-based approach from the knowledge of their transfer function, also considering the minimization of cell burden and failure rate caused by the multiple use of the same part in a circuit.

One of the main factors leading to unpredictable behaviour of synthetic circuits is cell burden [31]. The unnatural load caused by heterologously expressed genes can lead to transcriptional and translational resources depletion, exerting important global effects on the functioning of the designed circuit [31, 32]. Synthetic circuit designs aimed to reduce the metabolic load for the cell have been reported [33–36], in which systems with superior protein yield or functions were obtained. Experimental and in silico methods have also been recently proposed to analyze cell burden in synthetic circuits [35, 37–39]. The use of a constitutive expression cassette for a reporter gene, integrated in the bacterial chromosome, has been adopted as a real-time and in vivo burden monitor, to indirectly quantify the cellular resources limitation via microplate assay [35]. This methodology was demonstrated to be more sensitive than growth rate measurement for burden quantification. Other works have used the same approach, with the constitutive cassette assembled in plasmid, to study cell burden via modelling frameworks based on electronic engineering [39] and microeconomics [38]. Different mathematical models have been proposed for the analysis of protein expression in a limited resources context [37, 40, 41]. Such recently proposed models have been useful to identify the expression systems behaviours occurring when resources are limiting and cannot be trivially explained via simple Hill function-based activation/repression models [42]. However, such burden models still have shortcomings, e.g., they are unable to explain the possible separation of cellular resource pools among chromosome and plasmids (as suggested by Gyorgy et al. [38]) and the relationship between cell growth rate and resource pools is still lacking in such models, although it has been included in one recent study on dynamics of protein expression [43]. While most of the literature studies analyzed cell burden in non-interacting gene expression systems, a recent in silico study indicated that burden can largely affect the quantitative function of interconnected circuits, in which non-trivial activation and repression functions may emerge [37]. Such previously unexpected behaviour was confirmed by recent in vivo experiments involving a simple gene regulatory network, tested with two diverse regulatory gene RBSs and circuit copy numbers [44]. In the same work, an interaction graph-based theoretical framework was proposed to describe the effective interactions occurring among network modules, and eventually guide the design of circuits with different topologies [44].

The works mentioned above demonstrate the need of further steps towards the testing of a rigorous bottom-up approach in the design of interconnected synthetic circuits and they also highlight that cell burden is an important feature to be modelled in order to describe otherwise unpredictable outputs.

In this study, we analyzed synthetic transcriptional cascades in *Escherichia coli*, obtained upon interconnection of different inducible and repressible devices*.* To elucidate the reliability of currently available mathematical models applied to this circuit collection, we aim to test the predictive power of a widely used Hill function model and of one of the recently proposed models that considers cell burden due to resource limitation. Specifically, the latter considers Hill functions to describe specific interactions among circuit elements together with cell burden that modulates protein expression. These models are described in the Methods sections and will be referred to as *no-burden model* (NBM) and *burden model* (BM), respectively. The study presented in this work elucidates the feasibility boundaries of a bottom-up approach and the importance of taking into account cell burden in quantitative predictions.

## Results and discussion

### Circuits description

**Fig. 1:**
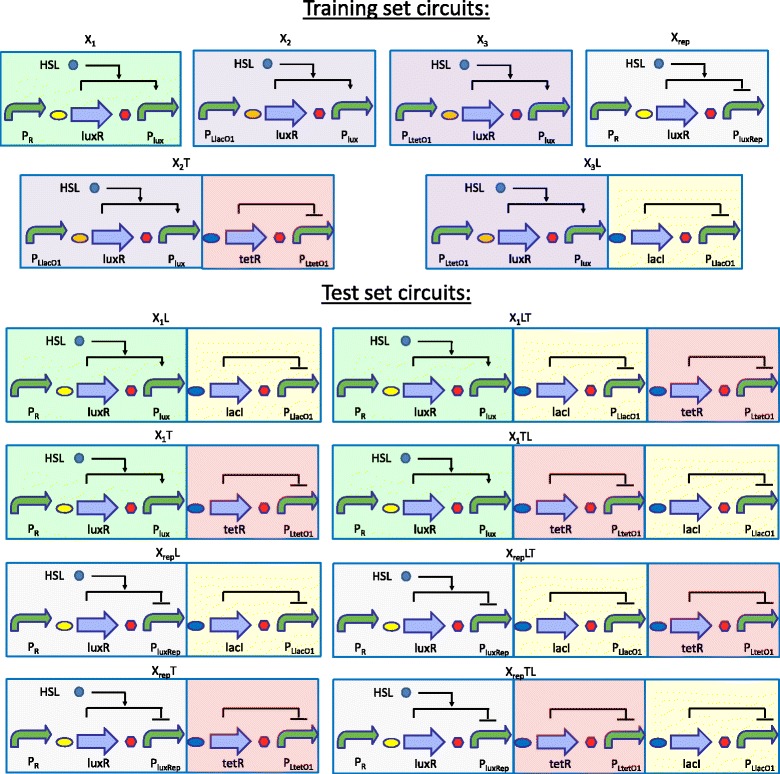
Collection of circuits analyzed in this study. All of them are available with an RFP expression system downstream of the output promoter (indicated in the text with the *r* suffix), and also with a GFP expression cassette driven by a constitutive promoter downstream (indicated in the text with the *rg* suffix, meaning that both RFP and GFP can be measured to quantify circuit output and cell burden, respectively). Curved green arrows represent promoters; straight violet arrows indicate coding sequences; red hexagons represent transcriptional terminators; ovals represent RBSs (BBa_B0030 yellow; BBa_B0034 orange; BBa_B0031 blue); circle represents HSL. Activation and repression are indicated as thin arrows. Block colour is consistent among the circuits

The circuits analyzed in this study are described in Fig. 1. Their design is based on the widely used lux, tet and lac systems elements, and the RFP and GFP reporter genes (see Additional file 1: Table S1 for a description of all the basic parts used). The circuits topology implements transcriptional cascades [25, 45] composed by an HSL-inducible or -repressible input block upstream of NOT gate blocks (none, one or two) connected in series. Finally, an RFP expression device is assembled downstream of the cascade to serve as detectable circuit output. All of them have been assembled in the low-copy pSB4C5 vector [46]. Input blocks include a constitutively expressed luxR gene with a strong RBS (BBa_B0030 or BBa_B0034) under the control of the P_R_, P_LtetO1_ or P_LlacO1_ promoter [47], and the wild-type inducible P_lux_ promoter [48] or the strongest member of a synthetic repressible promoter library [49], herein called P_luxRep_, downstream. The NOT gates include the tetR or lacI repressor gene, with a weak RBS (BBa_B0031), and their cognate repressible promoter P_LtetO1_ or P_LlacO1_, respectively, downstream [50]. The tetR and lacI genes both have an LVA fast-degradation tag for the translated protein [51].

One- to three-block cascades have been studied by using different combinations of these devices.

A collection of the same circuits has also been constructed with a reporter expression cassette downstream, composed by a GFP expression system driven by a constitutive promoter (BBa_J23100). This additional gene expression cassette will be referred to as *Monitor* cassette, and it will be adopted to quantify cell burden, as previously carried out [35, 38, 39].

The described circuits were divided into training and test sets (see Fig. 1). In particular, the X_1_, X_2_, X_3_ and X_rep_ configurations have been used as specific measurement system constructs for the characterization of input blocks, whereas X_2_T and X_3_L have been used to characterize individual NOT gates. The ones to characterize input blocks include the input device with RFP downstream. The ones to characterize NOT gates include a pre-characterized input device upstream, in order to tune the expression of tetR and lacI over a range of levels, and RFP downstream to measure the NOT gate block output. In the context of a bottom-up approach, the characterization of the circuits above was used to predict the behaviour of the test set circuits, which are composed by different combinations of the characterized blocks.

The used input devices provide homogeneous transcriptional output with no bimodal distribution of gene expression [19, 49, 52]. For this reason, the network topology used for the circuits in this work provides unimodal outputs at all the cascade levels.

### Data overview

Cascade output level at steady-state was measured as a function of HSL for all the circuits via RFP analysis (see Additional file 1: Figure S1). Considering circuits without Monitor, their RFP output span a wide range of values (>640 fold), with all the circuits showing a relevant output variation as a function of HSL concentration, from 3 fold (X_rep_TLr) to 141 fold (X_1_r). The growth rate of these recombinant strains span a 2.5 fold range (see Additional file 1: Figure S2). The quantitative behaviour of individual devices was consistent with previously published characterization data (see Additional file 1: Supplementary analysis of circuits data) [15, 49, 52–55].

According to the inducible or repressible behaviour of the constructed circuits that can be inferred from the individual blocks, all the 1- and 2-block circuits showed the expected logic behaviour. However, only two of the four 3-block circuits (X_1_LTr and X_rep_LTr) showed the expected logic output trend: the X_1_TLr and X_rep_TLr circuits, in which the output should be an increasing and decreasing function of HSL, respectively, showed a clear decreasing output (X_1_TLr), and an increasing and then decreasing (X_rep_TLr) output. Cell resource limitation may give rise to such qualitatively unpredictable behaviour, in which the logic activation and repression rules were affected by hidden interactions caused by cell burden [37]. For this reason, analysis of circuits in presence of a burden measurement system was carried out.

Considering circuits with Monitor cassette, GFP was analyzed (see Additional file 1: Figure S3), in addition to RFP and growth rate (see Additional file 1: Figures S1-S2), and used to indirectly measure cell burden. The RFP output and growth rate are highly similar to the ones of the circuits without Monitor, suggesting that the Monitor itself does not provide relevant burden to the cell (correlation value of 0.99 and 0.83, respectively, see Additional file 1: Figure S4).

In presence of a monitor cassette, a strong correlation between growth rate and GFP was previously observed, caused by growth rate decrease in presence of cell burden [35]. By contrast, here a statistically significant but very low correlation (0.27) was observed (see Additional file 1: Figure S5). Considering individual circuits, only three of them (X_2_Trg, X_1_Trg and X_1_TLrg) showed a statistically significant growth rate-GFP correlation (see Additional file 1: Table S2 and Figure S6), with the 0.41, 0.72 and 0.84 values, respectively. These constructs have in common a highly expressed TetR repressor, while in the other circuits its transcription is driven by weaker promoters. Consistently, these three circuits are also characterized by the lowest GFP levels among the tested circuits (see Additional file 1: Figure S3). Such data suggest that a correlation between growth rate and GFP can be detected only in the circuits causing the highest cell burden, while in other recombinant strains no significant growth rate-GFP correlation could be seen, even though GFP and growth rate showed large variations and GFP exhibits a clear HSL-dependent trend.

In previous studies of circuits including single non-interconnected expression cassettes, RFP and GFP also showed strong correlation because Monitor levels decrease when the expression of a second protein is triggered, due to resource allocation [38, 39]. The same negative strong correlation can be seen here for the four input devices (see Additional file 1: Table S2 and Figure S7). This trend cannot be observed for the other circuits, which include the regulated expression of different proteins, whose expression, together with the one of RFP, may provide a burden for the cell and give rise to complex RFP-GFP relationships.

The illustrated inter-relationships among RFP, GFP and growth rate confirm the usefulness of a burden monitor to measure cellular capacity instead of typically used growth rate measures. In fact, the output of a monitor cassette can not only provide an early cellular burden signal that precedes a growth rate decrease in dynamic experiments, as previously described [35], but also a more sensitive measure of cellular capacity, demonstrated by the clear RFP-GFP negative trend for the input devices (see Additional file 1: Figure S7), not reflected by growth rate changes (see Additional file 1: Figures S1-S3).

### Circuit predictability with the no-burden model

**Fig. 2:**
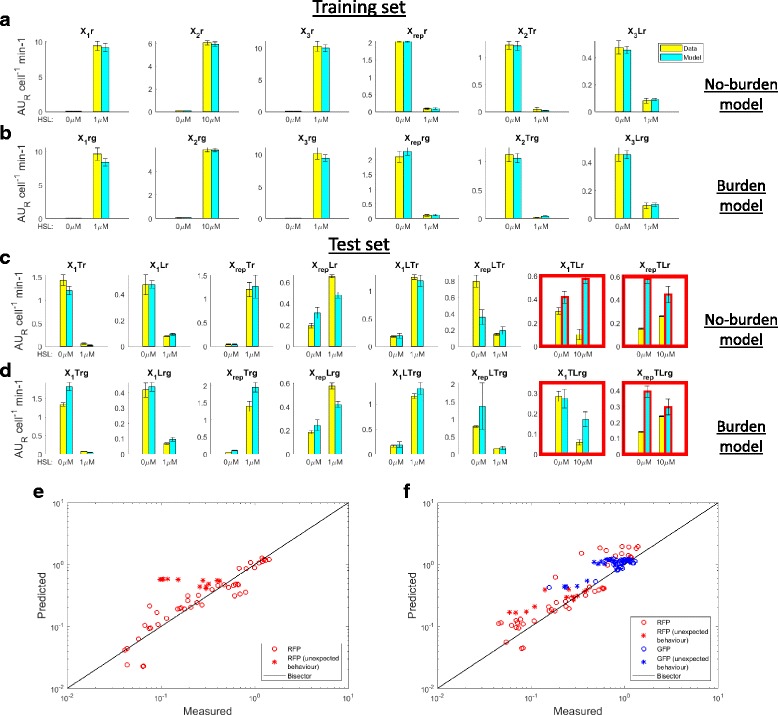
Overall prediction performance by the two models analyzed in this work. **a**-**d** Logic behaviour of the circuits in terms of RFP output level in vivo (yellow bars) and in silico (cyan bars) in absence of HSL and at the maximum HSL concentration tested. Results are shown for training set (**a**-**b**) and test set circuits (**c**-**d**), considering NBM (**a**, **c**) and BM (**b**, **d**). Red squares surrounding the sub-panels indicate a circuit configuration with unexpected in vivo behaviour. Red edges in the in silico-predicted output bars indicate that the model is not able to predict the observed logic behaviour of the circuit. Yellow bars represent the average output value and error bars represent the 95% confidence intervals of the mean. Cyan bars represent the median predicted value and error bars represent the 95% confidence intervals calculated via Monte Carlo simulations. **e**-**f** Measured output of the circuits at all the HSL concentrations tested in this work plotted against the values predicted by the NBM (**e**) and BM (**f**). Red and blue points represent RFP and GFP output, and are expressed as AU_R_ cell^−1^ min^−1^ and AU_G_ cell^−1^ min^−1^, respectively. Asterisks correspond to the data of the two circuits showing unexpected in vivo behaviour, while circles correspond to the data of all the other test set circuits. The solid line is the bisector line. Each point represents the average value of the in vivo measured condition, versus the median value of the corresponding model prediction

The data from the training set circuits were fitted with the NBM (see Methods section). A prediction performance summary is reported from a Boolean logic and a quantitative point of view (see Fig. 2b and e, respectively). The logic behaviour of all the circuits is accurately captured for all the training set circuits (Fig. 2a) and for all except two test set circuits: X_1_TLr and X_rep_TLr showed an unexpectedly non-increasing and non-decreasing HSL-dependent output, anticipated above, that was not predicted by the model (see Fig. 2b). The overall quantitative predictions on test set circuits showed a 0.88 correlation coefficient (see Fig. 2e).

The NBM fits the training set data accurately (see Additional file 1: Figure S8), and the estimated parameter values showed a relatively contained uncertainty (see Table 1).

**Table 1:** Parameters description and estimated values

Parameter	Units	Estimated value (NBM, training set)	Estimated value (BM, training set)	Estimated value (BM, via simultaneous fitting on all circuits)
α_X1_	AU_R_ cell^−1^ min^−1^	14.63 (3%)	36.17 (7%)	24.33 (4%)
K_X1_	nM	4.16 (9%)	5.39 (9%)	6.71 (6%)
η_X1_	-^*^	1.42 (3%)	1.51 (2%)	1.19 (2%)
δ_X1_	AU_R_ cell^−1^ min^−1^	0.14 (3%)	0.16 (3%)	0.2 (2%)
α_X2_	AU_R_ cell^−1^ min^−1^	9.06 (1%)	26.76 (3%)	20.36 (3%)
K_X2_	nM	15.06 (7%)	17.26 (6%)	31.39 (7%)
η_X2_	–	1.24 (3%)	1.25 (3%)	0.97 (2%)
δ_X2_	AU_R_ cell^−1^ min^−1^	0.14 (2%)	0.26 (5%)	0.18 (5%)
α_X3_	AU_R_ cell^−1^ min^−1^	15.81 (3%)	36.9 (5%)	35.67 (5%)
K_X3_	nM	4.37 (16%)	7.64 (17%)	8.9 (14%)
η_X3_	–	1.45 (6%)	1.41 (6%)	1.34 (6%)
δ_X3_	AU_R_ cell^−1^ min^−1^	0.16 (5%)	0.18 (3%)	0.19 (2%)
α_Xrep_	AU_R_ cell^−1^ min^−1^	2.85 (1%)	4.6 (4%)	8.22 (2%)
K_Xrep_	nM	6.67 (12%)	5.26 (11%)	1.86 (5%)
η_Xrep_	–	1.32 (12%)	1.21 (9%)	0.86 (2%)
δ_Xrep_	AU_R_ cell^−1^ min^−1^	0.13 (17%)	0.22 (14%)	0.09 (15%)
α_T_	AU_R_ cell^−1^ min^−1^	3.1 (1%)	3.45 (3%)	4.56 (2%)
K_T_	AU_R_ cell^−1^	6.47 (5%)	15.6 (7%)	6.92 (2%)
η_T_	–	1.59 (7%)	8.28 (31%)	2.57 (2%)
δ_T_	AU_R_ cell^−1^ min^−1^	0.03 (19%)	0.22 (3%)	0.21 (2%)
α_L_	AU_R_ cell^−1^ min^−1^	0.63 (6%)	0.56 (9%)	0.76 (2%)
K_L_	AU_R_ cell^−1^	56.39 (17%)	52.13 (22%)	34.92 (2%)
η_L_	–	1.91 (16%)	1.93 (42%)	1.93 (3%)
δ_L_	AU_R_ cell^−1^ min^−1^	0.11 (26%)	0.22 (19%)	0.22 (3%)
Σ_Xλ_	–	NA^*^	0.2 (8%)	0.36 (2%)
Σ_Xlac_	–	NA	1 (6%)	0.56 (2%)
Σ_Xtet_	–	NA	0.07 (23%)	0.12 (11%)
J_RFP_	AU_R_^−1^ cell min	NA	0.04 (3%)	0.04 (3%)
J_tet_	AU_R_^−1^ cell min	NA	0.07 (15%)	0.31 (2%)
J_lac_	AU_R_^−1^ cell min	NA	0.01 (13%)	0.01 (7%)
S_m_	AU_G_ cell^−1^ min^−1^	NA	1.75 (2%)	1.75 (2%)

*NA: not applicable; −: dimensionless

**Fig. 3:**
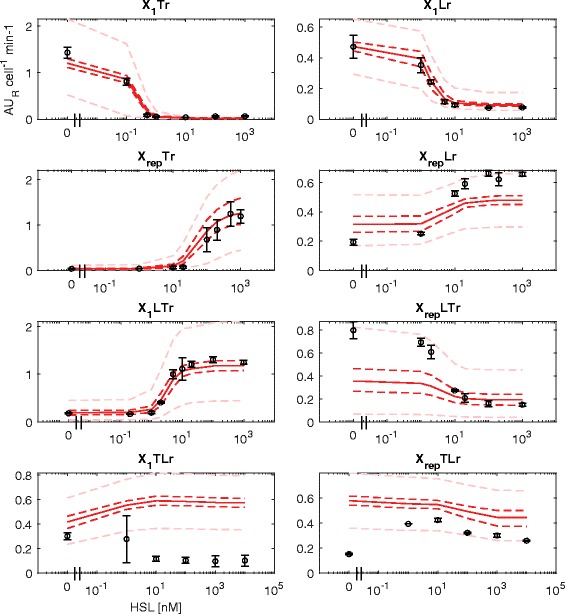
NBM prediction of the measured HSL-dependent output in all the test set circuits without Monitor cassette. Circles represent the average measured value and error bars represent the 95% confidence intervals of the mean. Solid line represents the median predicted output of the model calculated via Monte Carlo simulations for each HSL concentration tested. Dashed dark red lines are the 95% confidence bands of the output distribution. Dashed light red lines are the 95% confidence bands of the output distribution calculated after multivariate sensitivity analysis

Test set data could be accurately predicted by the identified model in the X_1_Tr, X_1_Lr, X_rep_Tr and X_1_LTr constructs (see Fig. 3). The X_rep_Lr and X_rep_LTr showed qualitatively correct predictions, but they underestimated the experimental data at maximum output level by up to 2 fold (see Figs. 2 and 3). On the other hand, as expected, the two remaining circuits (X_1_TLr and X_rep_TLr) did not show a correct prediction even qualitatively: a simple Hill function-based model is not able to describe their observed HSL-dependent RFP output (see Fig. 3).

To consider the effect of parameter uncertainty on the output prediction, uncertainty was propagated via Monte Carlo approach during the fitting and simulation procedure (see Methods section). In the training set, the resulting confidence bands of circuit outputs were very narrow, demonstrating a low uncertainty in model output, given the distribution of the estimated parameters (see Additional file 1: Figure S8). In the test set, the uncertainty of parameter values does not considerably affect many of the circuits: only X_rep_Tr and X_rep_LTr show relevant confidence bands around the central tendency value (see Fig. 3). Steep and sensitive genetic switches, i.e., biological devices in which the transfer function shows a steep response and starts increasing (or decreasing) for very low values of its input, have been proved to promote high-entity noise propagation throughout cascades of interconnected devices [56, 57]. As a consequence, in the latter situation the output curve is sensitive to small variations of parameters and activity of the input block. In the analyzed circuits, the TetR-based block is a highly sensitive switch, since even a small activity of the upstream block can result in an output value that is significantly lower than its maximum. This is demonstrated by the P_LtetO1_ output that, in presence of an upstream block in the off-state, is considerably lower than in absence of it (see Additional file 1: Figure S9). Conversely, the LacI-based block exhibits a similar output value in presence or absence of an upstream device in the off-state (see Additional file 1: Figure S9). The described situation may explain the large output uncertainty of X_rep_Tr and X_rep_LTr for high RFP levels (see Fig. 3). To confirm this effect on variability, univariate sensitivity analysis was carried out on the δ parameters of all the used devices, considering plausible variability range values for such parameters (see Methods section). Results showed that a relatively small variation of the basic activity of promoters was sufficient to cause high variability in the output curves in all the devices containing P_LtetO1_ as output promoter (X_1_Tr, X_2_Tr, X_rep_Tr, X_1_LTr and X_rep_LTr, see Additional file 1: Figure S10).

The results described above suggest that the robustness of the quantitative behaviour of the analyzed circuits can be low in some cases, due to relevant output variations in response to small variations of the parameters. Univariate sensitivity analysis was also carried out on the other three parameters of the Hill equation describing the devices transfer functions, α, K and η, to understand their effect on circuit outputs. The results, reported in Additional file 1: Figure S11-S13, showed that a variation of K and η could explain the experimental output of X_rep_LTr within confidence bands, but not the one of X_rep_Lr, while the variation of α is able to capture the output of both X_rep_LTr and X_rep_Lr. On the other hand, as expected, the parameter variations applied during sensitivity analysis could not describe the experimental output of X_1_TLr and X_rep_TLr, even by allowing the variation of all the four Hill function parameters (see Methods section and Fig. 3).

Evolutionary instability issues, such as mutations occurring in the genes or regulatory parts of the circuits, may cause alterations in their output [58]. To evaluate if the output trend of X_1_TLr and X_rep_TLr was due to such alterations, phenotypic and genetic stability was assayed via specific experiments (see Additional file 1: Figure S14). The on- and off-state output of both circuits were found to be reversible, i.e., cultures could reproducibly change RFP output level from low to high upon induction or de-induction, depending on the circuit (Additional file 1: Figure S14). Only X_1_TLr showed stability mutants occurring at high HSL concentrations (10 μM), but not at intermediate ones, although the RFP output decreases also in presence of 10 nM of HSL (Additional file 1: Figure S14). As anticipated above (and confirmed later in this work), TetR expression represents a burden for the cell, compared to the other proteins in the circuit, and this may explain the observed, yet low, instability occurring at high TetR synthesis levels. The X_rep_TLr circuit, on the other hand, did not show mutants. The output reversibility and the reduced presence of stability mutants only in one circuit and condition suggest that the unexpected RFP output is not due to evolutionary instability. Another issue might be enzymatic queuing, in which protein degradation complexes become a limiting resource and causes a slower degradation of all the proteins including the same specific degradation tag [59]. Although the X_1_TLr and X_rep_TLr circuits both include two proteins (TetR and LacI) with the same fast-degradation tag, simple in silico simulations showed that queuing effect could not explain the observed RFP output (see Additional file 1: Figure S15).

In summary, the NBM is able to successfully fit the experimental data from the training set and, by applying small variations to some of the model parameters, to quantitatively predict the output of all the set circuits with expected logic behaviour. On the other hand, the output of the two test set circuits that show unexpected behaviour was not captured by the NBM in any of the in silico experiments.

The experimental data of RFP output coming from the circuit collection with the Monitor cassette was also fitted with the NBM and analogous conclusions can be drawn (see Additional file 1: Figure S16).

### Circuit predictability with the burden model

Among the available models describing circuits output considering cell burden [37, 40, 41], we selected the one proposed by Qian et al. [37] (see Methods section) that was also adopted in other works [38, 39]. This model includes a low number of burden-related parameters, i.e., one for each gene in the circuit, while the other models, although successful in the in silico study of different situations [35, 40, 41], required the estimation or assumption of a larger number of parameters. The model by Qian et al. can be integrated into a simple Hill function model by introducing a protein synthesis-dependent factor, which has a global negative effect on the protein expression of all the circuit. The weight of each protein synthesis term quantifies the contribution of each circuit module to the global cell load, and has been previously used as a mechanistic model-derived lumped parameter measuring resource usage [37].

Analogously to what was performed for the NBM, the data from the training set circuits were fitted with the BM, by considering both RFP and GFP, representing the circuit output and the burden measures, respectively, and the output of test set circuits was finally predicted (see Fig. 2D and F for an overview of the logic and quantitative prediction performance).

Among the training (see Fig. 2b) and test set circuits (see Fig. 2D), only X_rep_TLr shows a non-correct logic behaviour prediction, while all the other circuit configurations could be captured. In particular, it is worth noting that the BM is able to predict the output of X_1_TLr, which could not be predicted by the NBM.

The training set data were fitted by the BM with reasonable accuracy (see Additional file 1: Figures S17-S18). In particular, the model showed excellent quantitative accordance with RFP experimental data (see Additional file 1: Figure S17), as it was observed above for the NBM; GFP data were all well fitted by the BM, except X_rep_rg and X_3_Lrg, for which the model showed a slightly lower descriptive capability to capture the measured data than for the other circuits (see Additional file 1: Figure S18).

The estimated parameter values showed significant deviations from the ones obtained via NBM (see Table 1). The most remarkable differences can be observed for the α parameter values of almost all the devices. These values are, in general, higher when estimated via the BM. This trend was expected, since in a limited cell resources framework the devices are globally burdened, and the estimated α values are linked to the maximum achievable activity, which may not be reached in any of the tested conditions [37]. For instance, the X_1_rg device shows a 2.5 fold difference in the α values between NBM and BM, meaning that the observed activity at full induction reached by the P_lux_ promoter in this device is much lower than maximum attainable one, which was 2.5 fold higher, due to the high RFP expression rate in this induction condition. A lower fold change is observed for devices characterized by lower activity in the on-state (e.g., X_rep_rg), since the α values estimated via the NBM are close to the maximum attainable ones, estimated by the BM. In addition to the Hill-related parameters, the BM includes resource usage parameters for each gene in the circuit [37]. The estimated values of the LuxR contribution to cell burden (Σ_Xλ_, Σ_Xlac_ and Σ_Xtet_, corresponding to the expression systems driven by P_R_, P_LlacO1_ and P_LtetO1_, respectively) showed that LuxR expression alone in the input devices causes cell burden and decreases circuit output by up to 50%, with the cassette driven by P_LlacO1_ giving the highest burden and the one driven by P_LtetO1_ giving the lowest one (see Table 1). This effect can be observed in the GFP output curves of the four input blocks in absence of HSL (see Additional file 1: Figure S18). In these four circuits in this condition, RFP expression is negligible and the only protein having a significant contribution to cell burden is LuxR; as a result, GFP output level is inversely correlated with the corresponding Σ value. This result was unexpected, since P_LlacO1_ has a lower activity than P_LtetO1_ in the used chassis, and a lower burden value for it was expected (given identical RBSs upstream of luxR). The estimated resource usage parameter values of RFP, TetR and LacI (*J*_*RFP*_, *J*_*tet*_ and *J*_*lac*_, respectively) enable to conclude that, with the used RBSs, TetR causes the highest cell load, while LacI the lowest one. Such values can be useful to evaluate the working boundaries in the bottom-up design of reduced-burden circuits, as demonstrated previously via different approaches [35].

**Fig. 4:**
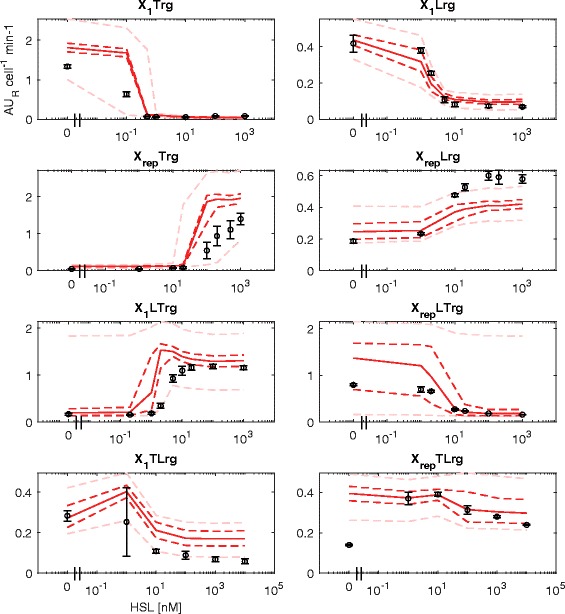
BM prediction of the measured HSL-dependent RFP output in all the test set circuits with Monitor cassette. Circles represent the average measured value and error bars represent the 95% confidence intervals of the mean. Solid line represents the median predicted output of the model calculated via Monte Carlo simulations for each HSL concentration tested. Dashed dark red lines are the 95% confidence bands of the output distribution. Dashed light red lines are the 95% confidence bands of the output distribution calculated after multivariate sensitivity analysis

**Fig. 5:**
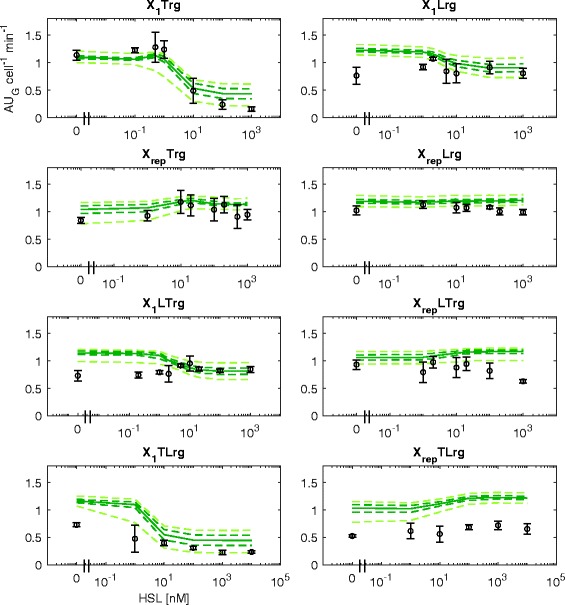
BM prediction of the measured HSL-dependent GFP output in all the test set circuits with Monitor cassette. Circles represent the average measured value and error bars represent the 95% confidence intervals of the mean. Solid line represents the median predicted output of the model calculated via Monte Carlo simulations for each HSL concentration tested. Dashed dark green lines are the 95% confidence bands of the output distribution. Dashed light green lines are the 95% confidence bands of the output distribution calculated after multivariate sensitivity analysis

Quantitative prediction results on the RFP output of test set circuits showed that the overall performance of the BM (correlation coefficient of 0.87, see Fig. 2F) is analogous to the one of the NBM. Considering individual circuits, X_1_Lrg, X_rep_LTrg and X_1_TLrg showed a good prediction with data consistent with the confidence bands of the model (see Fig. 4). Importantly, X_1_TLrg was one of the two circuits whose output could not be correctly predicted by the NBM; the other circuit (X_rep_TLrg), however, still behaved unpredictably. A slight over- (X_1_Trg and X_rep_Trg) or under-estimation (X_rep_Lrg) of the experimental data maximum output level was observed for three circuits, with an error up to 1.4 fold. The predicted output of X_1_LTrg showed a slightly anticipated switch point and an over-elongation at intermediate HSL concentrations that is not observed in experimental data. Considering the Monitor output of the same circuits (see Fig. 5), GFP showed an overall over-estimation of the experimental data, with a lower prediction performance than RFP (0.71 correlation coefficient, see Fig. 2F), suggesting that additional modelling work is needed to improve the predictive capability of burden-related models.

As it was carried out for the NBM, sensitivity analysis was performed. Results are shown for a multivariate sensitivity analysis (see Fig. 4-5). As expected, confidence bands are higher than for the NBM, since the BM has more parameters that can vary in a multivariate fashion. Results showed that all the RFP data can be explained by confidence bands, except X_rep_TLrg, leading to analogous conclusions drawn for the NBM: parameter variations of plausible entity can capture all the experimental data except for circuits showing qualitatively inconsistent predictions.

In summary, the BM does not improve the quantitative prediction performances of the analyzed circuits with expected behaviour, over the NBM. However, it correctly predicted the output of one of the two circuits with unexpected output behaviour, not predicted by the NBM, and, in addition, enabled the estimation of burden-related parameters that support the rational design of synthetic circuits.

### Model fitting using all the available experimental data

The BM was also identified by using all the available data of the training and test sets, in order to demonstrate the descriptive capability of the model to simultaneously fit all configurations, considering both RFP and GFP data as before (Additional file 1: Figures S19-S20). The NBM was used to fit the RFP experimental data as a term of comparison, but its descriptive capability was significantly worse than the one of the BM (Additional file 1: Figure S19). Model comparison demonstrated a significantly higher fitting performance for the BM (LR test, *p*-value <0.05). The parameter estimates resulting from the BM fitting are reported in Table 1. Their values confirm the conclusions about burden contribution levels for all the circuit proteins, since the Σ and J parameters have the same ranking as before, despite a large variation was observed for some of them (e.g., J_tet_ which was >4 fold higher than before).

Fitting results in Additional file 1: Figures S19-S20 show that the output of some configurations are over- or under-estimated up to 1.5 and 2.1 fold for RFP and GFP, respectively, although the outputs of all circuits is qualitatively captured. Parts activity variation upon interconnection, an open problem in synthetic circuit design, can explain the observed changes between individual configurations. Specifically, despite cell burden modelling can explain some unexpected phenomena in bottom-up designed circuits, other context-dependent effects still have to be quantitatively elucidated, e.g., promoter transcription variation caused by diverse flanking DNA sequences in different configurations [25].

The obtained results suggest that the modelling of cell burden significantly improves circuit output description capability, but the context-dependent behaviour of the assembled devices must be taken into account in future studies to predict new designed configurations more accurately.

### Fixing non-functional cascades via rational design

**Fig. 6:**
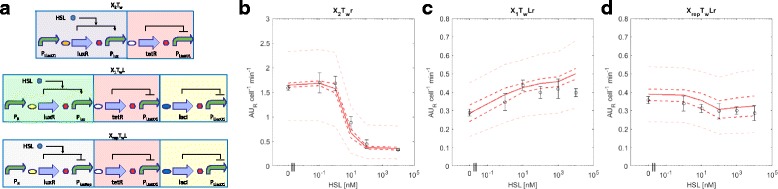
Analysis via NBM of the measured HSL-dependent output in the re-designed circuit variants, without Monitor cassette. **a** Circuit description. All of them are available with an RFP expression system downstream of the output promoter (indicated in the text with the *r* suffix). Symbols are described in Figure 1, except white ovals that represent a weak RBS (BBa_B0033), used in these circuits to decrease tetR expression. **b** Fitting of the training set circuit X_2_T_w_r. Estimated parameters were: α_T_ = 3.03 (4%), K_T_ = 63.14 (17%), η_T_ = 0.99 (10%), δ_T_ = 0.02 (>100%), where symbols have the same meaning as in Table 1 and CVs are reported in brackets. **c-d**) Prediction of the test set circuit X_1_T_w_Lr and X_rep_T_w_Lr, respectively. In panels B-D, circles represent the average measured value and error bars represent the 95% confidence intervals of the mean. Solid line represents the median output of the model calculated via Monte Carlo simulations for each HSL concentration tested. Dashed dark red lines are the 95% confidence bands of the output distribution. Dashed light red lines are the 95% confidence bands of the output distribution calculated after multivariate sensitivity analysis

To further demonstrate the usefulness of BM in the rational design of circuits, we designed and constructed new variants of the two cascades with unexpected behaviour to correct their HSL-dependent logic function. Based on the estimated resource usage parameters via BM (see Table 1), tetR was identified as the gene causing the highest load among the three regulated modules. For this reason, X_1_TLr and X_rep_TLr were mutagenized to decrease the translation efficiency of tetR, obtaining X_1_T_w_Lr and X_rep_T_w_Lr (Fig. 6a). Analogously, the training set circuit X_2_Tr was mutated, obtaining X_2_T_w_r, to enable the learning of the new tet-based NOT gate transfer function (Fig. 6a). The use of a weaker RBS (BBa_B0033 instead of BBa_B0031) upstream of tetR successfully modified the individual NOT gate transfer function (Fig. 6b), resulting in a less sensitive switch, as indicated by the *K*_*T*_ parameter that increased by 10 fold. Cascades with this modified tet-based NOT gate are expected to exert a lower cell load than their previous design when tetR is overexpressed, thereby restoring the correct functioning of the interconnected gates. Experimental results showed that this RBS change yielded circuits with expected increasing (X_1_T_w_Lr) and decreasing (X_rep_T_w_Lr) behaviour as a function of HSL (Fig. 6c-d). In addition, considering all the HSL concentrations tested, the new circuits had about 2 fold higher growth rate than the previous ones (data not shown). The RFP output of the realized circuits was also accurately predicted by the NBM, using the same training set as above (see Fig. 1) except X_2_T_w_r that was used instead of X_2_Tr (Fig. 6c-d). This result demonstrated that, after the attenuation of the main burden source, the two circuits with previously unexpected behaviour could be not only fixed in terms of qualitative behaviour, but also their quantitative HSL-dependent output could be successfully captured via traditional NBM.

## Conclusions

Recently proposed in vivo and in silico methodological approaches have been adopted to face the long-standing issue of biological devices predictability for the bottom-up design of synthetic circuits. Namely, a reporter expression cassette was used to quantify cell load and a mathematical model, which explicitly describes global burden-related effects on protein synthesis levels, was adopted for predictions. A set of ad-hoc constructed genetic circuits implementing transcriptional cascades was used as a testbed by following a rigorous bottom-up design process, including the learning of individual modules function (using a training set) and the evaluation of model predictions on a previously unseen circuit collection (using a test set).

From a qualitative point of view, considering the inducible/repressible behaviour of the circuits, the used model systems collection included circuits exhibiting expected HSL-dependent trend and a small set of circuits showing apparently unexpected outputs. The circuits spanned a wide range of RFP levels, corresponding to circuit output, and also showed diverse growth rates and GFP levels, indicating a variation of cell burden for different circuits and HSL inducer concentrations. Such statistics suggest that the considered collection includes sufficient variations in the observed variables to test the model descriptive and predictive capabilities.

A model-free correlation analysis of the measured data showed a strong negative correlation between GFP and RFP levels in the 1-block circuits, including only the input module with RFP downstream. This was expected, since high RFP expressions cause an increase of cell burden [38, 39]. Circuits with more than one block, on the other hand, did not show such trend, since the entity of cell load is expected to be not only RFP-dependent, but also function of the expression of the other circuit-borne proteins. On the other hand, the previously observed strong correlation between GFP (expressed via chromosomal constitutive cassette) and growth rate [35] was not observed in our data, which showed a weak correlation. This difference could be due to several factors, discussed below. By analyzing only the circuits exhibiting the lowest GFP values (corresponding to higher cell burden), their correlation with growth rate is relevant, while for the others it is non-significant. Circuits exhibiting a lower cell burden did not show a relevant variation in growth rate, but a clear HSL-dependent GFP trend could be observed. These results suggest that a GFP-growth rate correlation can be only observed if the circuits are affected by a relevant cell burden, and that the use of a constitutively expressed reporter protein has a clear superior performance, in terms of sensitivity, over the traditional use of growth rate for cell burden monitoring. Moreover, in this work we analyzed a different genetic context (Monitor cassette placed in plasmid instead of chromosome), and used different experimental protocols (HSL addition and growth to reach the steady-state of intracellular species) and data analysis methods (growth rates computed over the whole exponential growth phase, and GFP computed as fluorescent protein synthesis rate per cell) than the previous work [35].

Two models were compared in this work: NBM and BM. While the former uses RFP and growth rate data, the latter also uses GFP to eventually estimate and exploit the burden-related resource usage parameters. Both models were able to accurately fit the data of the training set and their overall quantitative prediction capability was comparable. Nonetheless, the BM allows to predict the output trend of one non-functional circuit exhibiting an unexpected output which could not be predicted by the NBM. Indeed, the behaviour of this circuit can be successfully explained only by modelling cell load.

However, another circuit exhibiting an unexpected output trend could not be predicted even by the BM.

It is worth noting that the two circuits with unexpected logic behaviour analyzed in this study are not robust logic gates, since the difference predicted by both the NBM and BM between on-state and off-state is very low. In fact, despite the transfer functions of all the devices have a wide induction range (see Additional file 1: Figure S8-S9), their output range may not be entirely spanned in the interconnected configurations. In these two circuits, the transcriptional input provided to the lac-based NOT gate is predicted to exert a detectable but not tight repression on P_LlacO1_, thereby covering a small part of its available output range. Moreover, the on-state has a transcriptional output activity comparable with a medium-strength promoter and the off-state has a high basic activity. While software tools able to guide the design of robust functions have been proposed [30], the design of robust gates was beyond the scope of this work: we limited our study to the analysis and predictability of the qualitative and quantitative output observed from the interconnection of pre-characterized modules. The previously proposed software system also considers cell load by identifying the part configurations in which a relevant impairment of cell growth was observed. The BM used in our study could be a further support in the rational engineering of genetic circuits, also from the knowledge of the resource usage of all the involved proteins (see below).

The NBM and BM were also systematically compared in terms of descriptive capabilities. To carry out this task, all the available data were considered and fitted with the NBM and BM. The BM captures circuits output with higher accuracy than the NBM, which is unable to capture the experimental output exhibited by some of the circuits even qualitatively. An over- or under-estimation of RFP and GFP outputs, up to 1.5 and 2.1 fold, respectively, were still present; such fold change values are reasonably contained, and comparable to other studies focused on predictability [15, 22, 49]. Context-dependent variability might cause such variation. Previous studies proposed a linear model-based method to score the quality of part collection members, relying on the characterization of their activity in different genetic constructs [29]. Analogously, because of context-dependent variability of parts, the BM may be unable to explain all the variation observed in the experimental data, and the same devices measured in different context can show diverse activity. In case of simultaneous fitting, the estimated parameter values represent the average values that best describe parts behaviour considering all the circuits. On the other hand, if a parameter value is estimated on a single training set circuit, it represents the specific value of the analyzed circuit. In the training set circuits used in this study, all the model parameters have been estimated considering a single circuit, except the burden-related parameters, which were the result of a simultaneous fitting considering all the circuits including the protein causing resource usage.

The fitting of the training set data by the BM enabled to estimate resource usage parameters for the proteins involved in the cascades. With the used RBSs, TetR was found to cause more cell load than LacI (i.e., the other repressor used) and RFP. This resource usage ranking was confirmed through the parameter estimation results of the simultaneous fitting of all the available data (not only training set circuits) by the BM. The contribution of LuxR to cell load was estimated as a non-dimensional parameter lumping the product of resource usage parameter and maximum LuxR synthesis rate per cell, since, differently from the other circuit proteins, the latter was not estimated in this study. For this reason, in the present work the resource usage of LuxR cannot be directly compared with the other considered proteins.

Based on the resource usage estimation via BM, we finally fixed the two circuits with unexpected behaviour by decreasing the translation of the gene causing the main load to the cell. To this aim, a 10 fold decrease in tetR translation efficiency successfully restored a correct function for both circuits, and the NBM was able to accurately predict the observed output. The obtained results demonstrate the usefulness of BM in the identification of the modules causing excessive cell load and the successful utilization of resource usage knowledge to drive the rational re-design of predictable circuits.

Based on the cell burden measurements and modelling, in both a bottom-up and global fitting fashion, and on the subsequent study of low-burden variants, we enabled to confirm that the unexpected behaviour of the X_1_TL and X_rep_TL configurations was due to tetR overexpression, which caused excessive cell load. Despite tetR affects the two circuits by breaking their logic behaviour, it is worth noting that cell burden also affects other circuits without breaking their function. In particular, according to GFP measurements and BM predictions, tetR exerts a high load in the X_2_T and X_1_T configurations, even higher than in X_rep_TL. However, this affect is not visible, since the high-load condition persists when the circuit output is low, thereby masking any burden-induced effects on the expected logic function of these circuits.

Taken together, our results showed that the use of the BM has advantages over using the NBM, in terms of predictability of some configurations in bottom-up approach, descriptive power of circuit and Monitor output, estimation of load-corrected transfer function parameters and estimation of the resource usage parameters, which can support the rational design of circuits with predictable function.

However, several steps still need to be carried out to improve the predictability of models like the ones used in this study. For instance, here the growth rate was fixed in the model for each circuit and HSL concentration, without any effort to predict it from the specific circuits used. The knowledge of growth rate could affect protein dilution, and recombinant strains with different dilution rates may exhibit diverse quantitative circuit behaviours. The growth rate prediction task is hampered by its poor predictability as a function of GFP value by the Monitor. In addition, as it was anticipated in the background section, cell growth rate may have a relationship with the amount of resource pools (which are assumed to be constant in the BM used in this study). New models considering this aspect could give significant benefits in the description and prediction of experimental data from synthetic circuits, as well as improve the understanding of biological systems features.

Our results show the outcome achieved via bottom-up design process considering limited cell resources and demonstrate the need of further efforts to improve models for biological engineering, to disclose hidden interactions among biological systems elements.

## Methods

### Strains, reagents and cloning

The *E. coli* TOP10 (Invitrogen) strain was used as a host for cloning and quantitative assays. The strain was transformed by heat shock at 42 °C according to manufacturer’s instructions. LB medium was used during plasmid propagation. Antibiotics were always added to maintain plasmids in recombinant strains: ampicillin (100 mg/l), kanamycin (50 mg/l) or chloramphenicol (12.5 mg/l). Long-term bacterial stocks were prepared for all the engineered strains by mixing 750 μl of a saturated culture with 250 μl of 80% glycerol, and stored at −80 °C.

All the plasmids used in this study were constructed through BioBrick(TM) Standard Assembly [60] and conventional molecular biology techniques. As a result, standard DNA junctions (TACTAG upstream of coding sequences, TACTAGAG otherwise) are present between assembled parts. The BioBrick(TM) basic or composite parts used for DNA assembly were retrieved from the MIT Registry 2008–2011 DNA Distribution [61], except P_luxRep_, which was constructed in a previous study [49], and the weak BBa_B0033 RBS that was placed upstream of tetR via mutagenic PCR, replacing BBa_B0031.

DNA purification kits (Macherey-Nagel), restriction enzymes and T4 DNA ligase (Roche), Phusion Hot Start II PCR kit and T4 polynucleotide kinase (Thermo Scientific) were used according to manufacturer’s instructions. Plasmids were sequenced via the BMR Genomics DNA analysis service (Padova, Italy). Oligonucleotides for mutagenesis (REV_LUXWT: 5′-tttattcgactataacaaaccattttcttgcg-3′, REV_LUXREP: 5′-gctagcattatacctgtacgatcctacaggtg-3′, FWD_TET33: 5′- tactagagtcacacaggactactagatgtccagattagataaaagtaaag-3′) were obtained from Metabion International AG.

M9 supplemented medium (11.28 g/l M9 salts, 1 mM thiamine hydrochloride, 2 mM MgSO4, 0.1 mM CaCl2, 0.2% casamino acids and 0.4% glycerol) was used in quantitative experiments. HSL (#K3007, Sigma Aldrich) was dissolved in deionized water to prepare a 2 mM stock, stored at −20 °C.

### Circuits characterization

Fluorescence and absorbance of recombinant bacteria incubated in a microplate reader were measured over time as previously described [15, 49, 52]. Briefly, bacteria from a glycerol stock were streaked on a selective LB agar plate. After 16- to 20-h incubation at 37 °C, 1 ml of selective M9 was inoculated with a single colony. For strains expressing a repressor in absence of HSL, the inducer was added at this step, at a proper concentration, to allow them to reach a steady-state of intracellular proteins, and to avoid long dynamics due to repressor proteins degradation and dilution during the microplate assay. After 21-h incubation at 37 °C, 220 rpm, in an orbital shaker, cultures were 100 fold diluted in a final volume of 200 μl in a 96-well microplate. HSL (2 μl) was added when required, to reach the desired final concentration. Cultures were not placed in the external wells of the plate to avoid intensive evaporation during incubation. The microplate was incubated with lid in the Infinite F200 microplate reader (Tecan) and it was assayed via kinetic cycle: 15 s linear shaking (3 mm amplitude), 5 s wait, absorbance (600 nm) measurement, fluorescence measurements, 5 min sampling time. RFP and GFP fluorescence was measured with a gain of 80 with the 535/620 nm and 485/540 filter pairs, respectively. Control wells were always included, as described in the Data processing section, to measure the background of absorbance and fluorescence, and to provide internal control references for relative activity calculations. At least three biological replicates, starting from different colonies, were assayed for each strain.

### Data processing

Data analysis and graphs were carried out via Microsoft Excel and Matlab R2007b or R2017b (MathWorks, Natick, MA). Pairwise correlations and corresponding *p*-values, as well as correlation matrices, were computed via the Matlab *corr* function. Linear regression was carried out via the Matlab *regress* function.

Raw absorbance and red fluorescence time series were blanked by background subtraction as previously reported [15, 62] to obtain OD_600_ and RFP time series. Sterile medium and a non-fluorescent TOP10 culture were used as absorbance and red fluorescence background, respectively. Since a significant cell density-dependent autofluorescence was previously reported for GFP measurements with our experimental setup [63], green fluorescence was blanked via a different procedure: for each GFP-expressing strain, a control strain with identical circuit and HSL concentration, but without GFP expression cassette, was considered. The raw green (auto)fluorescence (GFP_auto_) vs OD_600_ characteristic (at least two biological replicates) was fitted via an exponential regression (${GFP}_{auto}(t)={\mathrm{e}}^{\mathrm{q}+\mathrm{m}\bullet {\mathrm{OD}}_{600}\left(\mathrm{t}\right)}$); this curve was used to estimate the green fluorescence background of a target culture, given its OD_600_ at each time point. The GFP_auto_ value was subtracted from the raw fluorescence of the target culture to obtain a signal proportional to the GFP level in the whole culture. Fluorescent protein synthesis rate per cell (S_cell_) was computed for each culture and fluorescent protein as ${S}_{cell}=\frac{dF}{dt}\bullet \frac{1}{O{D}_{600}}$ (where *F* is the RFP or GFP level in the whole culture) and it was averaged over the exponential growth phase (0.05 < OD_600_ < 0.18) [49]. The obtained values were divided by the average S_cell_ of a reference culture, constitutively producing RFP or GFP with the same expression system under the control of the BBa_J23101 promoter, yielding S_cell,norm_. Reference cultures for RFP and GFP have the BBa_J107029 and BBa_K173001 expression cassettes, respectively. When the growth rates of target strain and reference cultures are similar, S_cell,norm_ is equivalent to the Relative Promoter Unit value.

A strain only including the Monitor cassette was also considered (herein called *Monitor culture*) to estimate the GFP level without the cell load caused by the circuits. In this strain, a BBa_B0015 transcriptional terminator was assembled upstream of the Monitor cassette to enable GFP measurements with the BBa_J23100 promoter in the same surrounding context of all the circuits, in which this terminator is always present upstream of the Monitor cassette.

Growth rate was computed via linear regression of ln(OD_600_) vs time characteristic in the 0.05 < OD_600_ < 0.18 window [49, 63].

The inclusion of specific control strains without Monitor cassette in different conditions for target strain autofluorescence estimation was necessary because such background value was found to be not only OD_600_-dependent, but also growth rate-dependent (see Additional file 1: Figure S21-S22), and strains bearing circuits with or without Monitor have similar growth rates (see Additional file 1: Figure S4A). The background of the GFP reference culture and the Monitor culture was estimated using the RFP reference culture as control.

### Mathematical model description: No-burden model (NBM)

We considered models including Hill functions to describe activation and repression of proteins expression in the analyzed circuits. Intracellular protein levels were modelled via dynamic equations as previously performed in many works [20, 28, 30], assuming the steady-state of all the intracellular species in exponentially growing cells, and assuming no metabolic burden affecting the cells.

The level of a repressor protein (*P*_*j*_) in the NOT gate blocks is computed as:

1 $$ {P}_j=\frac{1}{\mu +{\gamma}_j}\cdotp {S}_j $$

where γ is the protein degradation rate due to the LVA tag, μ is the cell growth rate, which depends on recombinant strain and HSL concentration, and *S*_*j*_ is the *P*_*j*_ synthesis rate per cell, defined as:

2 $$ {S}_j={\delta}_j+\frac{\alpha_j}{1+{\left(\frac{K_j}{I_j}\right)}^{\pm {\eta}_j}} $$

where δ, α, K and η are the Hill equation parameters that characterize the upstream regulated promoter; in particular, δ is the basic expression rate in the off-state, δ + α is the maximum expression rate, K is the input (*I*) level corresponding to 50% of the expression rate range, and η is the Hill coefficient (positive if the upstream promoter is inducible, negative if repressible); finally, *I* is the function input, which can be a per-cell protein level (if the NOT gate has another NOT gate block upstream) or HSL concentration (if the upstream block is an input block). Growth rate is assumed to affect protein dilution rate due to cell doubling, but not all the other processes (e.g., transcription and translation).

The immature (i.e., non-fluorescent) reporter protein per-cell level (*R*) is computed as:

3 $$ R=\frac{1}{\mu +a}\cdotp {S}_j $$

where *a* is the fluorescent protein maturation rate and the other symbols have the same meaning as above. Finally, the mature reporter protein synthesis rate per cell (S_cell,norm_), which is the measured output of the circuits, is computed as [8]:

4 $$ {S}_{cell,\mathit{\operatorname{norm}}, RFP}=a\cdotp R $$

S_cell,norm,RFP_ is expressed as arbitrary units of RFP (AU_R_, if considering circuit output) per cell per time (AU_R_ cell^−1^ min^−1^). RFP per cell concentration is assumed to be proportional to its respective arbitrary units. To support the predictable interconnection of biological devices, inputs and outputs of all the circuit blocks need to be expressed with the same units [20]. To this aim, the regulated promoter of all the blocks is characterized in AU_R_ cell^−1^ min^−1^ units and the intracellular levels of all the proteins of the network can be expressed as AU_R_ cell^−1^. The basic underlying assumptions are that a promoter is able to drive any downstream-connected gene to the same activity-dependent expression level [28], and the resulting protein level is assumed to be proportional to the gene expression level [8]. Such assumptions enable to model and re-use different biological devices by expressing their activities in comparable units [20], in absence of context-dependent variation of parts function [64].

### Mathematical modelling in a limited resource context: Burden model (BM)

In a limited resource context, RNA polymerase and ribosome intracellular levels have also to be taken into account. The full model derivation procedure and assumptions are described in the work by Qian et al. [37]. Briefly, from a structural point of view, the only difference between the BM and the standard model based on Hill equation (NBM) is the presence of a denominator (*D*) that affects all the protein synthesis rates. Referring to Eq. 2, the protein synthesis rate term becomes:

5 $$ {S}_j=\frac{S_{\mathit{\max},j}}{D} $$

6 $$ {S}_{\mathit{\max},j}={\delta}_j+\frac{\alpha_j}{1+{\left(\frac{K_j}{I_j}\right)}^{\pm {\eta}_j}} $$

7 $$ D=1+{\sum}_{k=1}^M{J}_k\cdotp {S}_{\mathit{\max},k} $$

where *M* is the number of expressed proteins in the cell, *S*_*max,k*_ is the maximum achievable synthesis rate of the k-th expressed protein, and *J*_*k*_ is the related resource usage parameter, representing a measure of the burden caused by the k-th protein. This denominator includes not only the effect of the genes in the synthetic network, but also the ones of the organism. The sum of the organism gene contributions to the burden (*z*) can be assumed to be a constant circuit- and induction-independent term:

8 $$ z={\sum}_{k=1}^C{J}_k\cdotp {S}_{\mathit{\max},k} $$

where *C* is the number of expressed organism genes. The denominator *D* can be re-written as:

9 $$ D=1+z+{\sum}_{k=1}^Y{J}_k\cdotp {S}_{\mathit{\max},k} $$

where *Y = M-C* is the number of proteins expressed in the synthetic circuit. Being (1 + *z*) a constant term, *D* can be rescaled by dividing each term by (1 + *z*) to obtain $\widehat{D}$:

10 $$ \widehat{D}=1+{\sum}_{k=1}^Y{J}_k\cdotp \widehat{S_{\mathit{\max},k}} $$

where $\widehat{S_{\mathit{\max},k}}$ are the maximum achievable synthesis rates rescaled by (1 + *z*). In this case, all the synthesis rates and intracellular protein levels in the model are rescaled by this term. For this reason, the *P*_*j*_, *S*_*max*,j_ and *S*_*j*_in the BM can be interpreted as the protein level, maximum synthesis rate and actual synthesis rate relative to the endogenous resource usage term, 1 + *z*, without affecting their units or the functionality of the model. In the BM, the Hill equation represents the maximum achievable synthesis rate (Eq. 5–6) and has a different interpretation compared to the NBM, in which it represents the actual synthesis rate (Eq. 2). Since all the circuits analyzed with the BM contain the Monitor cassette, the constant contribution of GFP expression was included among the organism genes (although GFP expression was found to have a negligible contribution to cell burden, as described in the Results and Discussion section) without affecting the meaning of the described quantities.

The resource usage terms, *J*, are expressed in (AU_R_^−1^ cell min) units. However, the contribution to $\widehat{D}$ of non-regulated proteins in the circuits (i.e., LuxR in the input blocks) is herein expressed by the dimensionless constant term $\Sigma =J\cdotp \widehat{S}$.

Differently from the NBM, GFP expression is also modelled to enable the quantification of cell burden. The intracellular level (*G*) of immature GFP is computed as:

11 $$ G=\frac{1}{\mu +{a}_G}\cdotp \frac{S_m}{\widehat{D}} $$

where *a*_*G*_ is GFP maturation rate, *S*_*m*_ is the synthesis rate in the Monitor cassette (expressed as arbitrary units of GFP - AU_G_ - cell^−1^ min^−1^), and the other symbols have the same meaning as above.

Analogously to the RFP output, the Monitor cassette output is described as:

12 $$ {S}_{cell,\mathit{\operatorname{norm}}, GFP}={a}_G\cdotp G $$

In addition to the NBM assumptions, we further assume that RNA polymerase and ribosome levels are not affected by cell growth rate, and that cell burden and growth rate do not considerably affect the transfer function of input devices due to LuxR protein level variation [39]. The inclusion of these two phenomena would require a model relating growth rate and RNA polymerase/ribosome levels, and the explicit modelling of LuxR production and binding with HSL via a mechanistic model, such as the one proposed by Carbonell-Ballestero et al. [53]. Both interventions are beyond the scope of this work and can be topic of additional modelling studies.

### Model fitting and analysis

Matlab R2007b was adopted for model fitting and analysis. Fitting was performed using the weighted least squares method via the *lsqnonlin* function. For each data point at a given HSL concentration, the weight of the i-th squared residual was set to ${w}_i=\frac{1}{\sigma_i^2}$, where *σ*_*i*_ is the standard deviation of all the biological replicates at the given HSL concentration. Biological replicates showed a relatively low variability in terms of growth rate (average CV of 12%, with a range of 1–36%); for this reason, the growth rate of recombinant strains was set to the average growth rate value at a given HSL concentration.

Unless differently stated, the NBM and BM were fitted sequentially: in the NBM, the Hill parameters of the four input blocks were first learned individually; then, the Hill parameters of the two NOT gates were learned individually, by setting the Hill parameters of their input devices to the values estimated in the first learning step. In the BM, the four input blocks were first simultaneously fitted to estimate the respective Hill parameters and the burden-related parameters, i.e., *J*_*RFP*_, Σ_*Xλ*_, Σ_*Xlac*_ and Σ_*Xtet*_; then, the Hill parameters of the two NOT gates, as well as their burden-related parameters, *J*_*tet*_ *and J*_*lac*_, were learned individually as before, by setting the other parameters to the previously estimated values.

Implicit equations, commonly occurring in the BM due to the presence of protein levels on the left and right hand side (see Eqs. 1, 5–7), were solved using a custom Matlab script implementing the fixed point method.

The NBM and BM were also fitted by using the data of all the circuits, and their parameters were all simultaneously estimated. In this case, the two models were compared via the Likelihood Ratio (LR) test, in which the log-likelihood value was computed as $LL=-\left({\sqrt{2\pi}}^{\ast}\sum \limits_i^N{\sigma}_i+\frac{1}{2}\sum \limits_i^N{r}_i^2\right)$ considering RFP data, where *N* is the number of data points and ${r}_i^2$ is the i-th weighted squared residual, assuming that experimental data are affected by uncorrelated Gaussian error with standard deviation *σ*.

Unless differently indicated, fixed values were used for the following parameters in all the fitting and simulation procedures: *γ*_*tet*_=0.0173 min^−1^ [51], *γ*_*lac*_=0.0533 min^−1^ [65], *a* = 0.0167 min^−1^ [29] and *a*_*G*_ = 0.0462 min^−1^ [29].

A Monte Carlo approach was adopted to estimate parameter uncertainty and to propagate it throughout the model fitting procedure. For each model fitting step, 10,000 synthetic datasets were created by adding Gaussian noise (with zero mean and variance ${\sigma}_i^2$) to the model prediction computed with the estimated parameters [49]. Negative data were set to zero. The fitting procedure was carried out for each dataset and a distribution of estimated parameters was obtained. In the stepwise procedure, parameter sets were randomly extracted from the previously obtained distribution instead of fixing them during the NOT gates model identification step, to properly propagate the uncertainty of parameter estimation to the downstream learning steps.

Univariate sensitivity analysis (i.e., performed on a single Hill parameter - δ, α, K or η - for all the blocks of a circuit) was carried out by following the Monte Carlo method illustrated above, but replacing the target parameter distribution with a Gaussian distribution with the same mean and CV = 25%. This variability was set to impose that the 95% confidence intervals of parameters (p) are 0.5*p and 1.5*p, which are reasonable context-dependent variability values seen in other studies (although larger variability can be observed in distribution tails [22]). Multivariate sensitivity analysis (i.e., performed on all the four Hill parameters in all the blocks) was carried out analogously, except that the Hill parameters were extracted from a multivariate Gaussian distribution, taking into account the correlation between parameter estimates.

Monte Carlo model simulations, aimed to predict the test set circuits output, were carried out by extracting parameter sets from the estimated distributions. Predictions were performed by fixing the growth rates to the experimentally measured values.

## Additional file

Additional file 1:
**Supplementary Notes.** Supplementary analysis of circuits data. **Supplementary Figures. Figure S1.** RFP output data for the cascade circuits tested in this work (training and test set) as a function of HSL concentration. **Figure S2.** Growth rate data for the cascade circuits tested in this work (training and test set) as a function of HSL concentration. **Figure S3.** GFP output data for the cascade circuits with Monitor cassette tested in this work (training and test set) as a function of HSL concentration. **Figure S4.** Growth rate (A) and RFP (B) comparison plots between strains without and with Monitor cassette at all the tested HSL concentrations for all the circuits (training and test set). **Figure S5.** Correlation between GFP and growth rate in all the strains (training and test set) with the Monitor cassette at all the tested HSL concentrations. **Figure S6.** Correlation between GFP and growth rate for each strain with the Monitor cassette at all the tested HSL concentrations. **Figure S7.** Correlation between GFP and RFP for each strain with the Monitor cassette at all the tested HSL concentrations. **Figure S8.** NBM fitting of the measured HSL-dependent output in all the training set circuits without Monitor cassette. **Figure S9.** NBM fitting of the two NOT gate characteristics as a function of the predicted per cell concentration of the TetR or LacI repressor. **Figure S10.** Univariate sensitivity analysis of the NBM by applying a variation on the δ parameter of the Hill functions. **Figure S11.** Univariate sensitivity analysis of the NBM by applying a variation on the α parameter of the Hill functions. **Figure S12.** Univariate sensitivity analysis of the NBM by applying a variation on the K parameter of the Hill functions. **Figure S13.** Univariate sensitivity analysis of the NBM by applying a variation on the η parameter of the Hill functions. **Figure S14.** Evolutionary stability of the X_1_TLr and X_rep_TLr circuits. **Figure S15.** Simulation of X_1_TLr and X_rep_TLr with the NBM for different values of γ_tet_ and γ_lac_ parameters. **Figure S16.** Fitting and prediction results for the NBM learned and simulated against RFP data of the circuits with the Monitor cassette. **Figure S17.** BM fitting of the measured HSL-dependent RFP output in all the training set circuits with Monitor cassette. **Figure S18.** BM fitting of the measured HSL-dependent GFP output in all the training set circuits with Monitor cassette. **Figure S19.** Results of fitting using all the available data (training and test set) using NBM and BM: RFP data. **Figure S20.** Results of fitting using all the available data (training and test set) using BM: GFP data. **Figure S21.** OD_600_, raw GFP and raw RFP values measured in culture, supernatant and pellet of three strains. **Figure S22.** Raw GFP autofluorescence depends on OD_600_ and also cell growth rate. **Supplementary Tables. Table S1.** Parts used in this study. **Table S2.** Statistics on circuits bearing the burden monitor. (PDF 2853 kb)

## Abbreviations

5′-UTR: 5′ untranslated region
BM: burden model
GFP: Green Fluorescent Protein
HSL: N-3-oxohexanoyl-L-homoserine lactone
NBM: no-burden model
OD_600_: optical density at 600 nm
PCR: polymerase chain reaction
RBS: ribosome binding site
RFP: Red Fluorescent Protein

## References

[1] Sauro HM (2008). Modularity defined. Mol Syst Biol.

[2] Del Vecchio D, Ninfa AJ, Sontag ED (2008). Modular cell biology: retroactivity and insulation. Mol Syst Biol.

[3] Pasotti L, Zucca S. Advances and computational tools towards predictable design in biological engineering. Comput Math Methods Med 2014; 2014:369681. doi: 10.1155/2014/369681. 10.1155/2014/369681 PMC4137594 25161694

[4] Schwille P (2011). Bottom-up synthetic biology: engineering in a tinkerer's world. Science.

[5] TK L, Khalil AS, Collins JJ (2009). Next-generation synthetic gene networks. Nat Biotechnol.

[6] Arkin AP (2013). A wise consistency: engineering biology for conformity, reliability, predictability. Curr Opin Chem Biol.

[7] Muers M (2013). Synthetic biology: quality and quantity. Nat Rev Genet.

[8] Kelly JR, Rubin AJ, Davis JH, Ajo-Franklin CM, Cumbers J, Czar MJ (2009). Measuring the activity of BioBrick promoters using an in vivo reference standard. J Biol Eng.

[9] Salis HM, Mirsky EA, Voigt CA (2009). Automated design of synthetic ribosome binding sites to control protein expression. Nat Biotechnol.

[10] Espah Borujeni A, Channarasappa AS, Salis HM (2014). Translation rate is controlled by coupled trade-offs between site accessibility, selective RNA unfolding and sliding at upstream standby sites. Nucleic Acids Res.

[11] Chen YJ, Liu P, Nielsen AA, Brophy JA, Clancy K, Peterson T, Voigt CA (2013). Characterization of 582 natural and synthetic terminators and quantification of their design constraints. Nat Methods.

[12] Cambray G, Guimaraes JC, Mutalik VK, Lam C, Mai QA, Thimmaiah T (2013). Measurement and modeling of intrinsic transcription terminators. Nucleic Acids Res.

[13] Hajimorad M, Gray PR, Keasling JD. A framework and model system to investigate linear system behavior in *Escherichia Coli*. J Biol Eng. 2011;5:3. 10.1186/1754-1611-5-3 PMC3110104 21510907

[14] Guido NJ, Wang X, Adalsteinsson D, McMillen D, Hasty J, Cantor CR (2006). A bottom-up approach to gene regulation. Nature.

[15] Pasotti L, Politi N, Zucca S, Cusella De Angelis MG, Magni P (2012). Bottom-up engineering of biological systems through standard bricks: a modularity study on basic parts and devices. PLoS One.

[16] Ceroni F, Furini S, Stefan A, Hochkoeppler A, Giordano E. A Synthetic post-transcriptional controller to explore the modular design of gene circuits. ACS Synth Biol. 2012;1:163–71. 10.1021/sb200021s 23651154

[17] Jayanthi S, Nilgiriwala K, Del Vecchio D (2013). Retroactivity controls the temporal dynamics of gene transcription. ACS Synth Biol.

[18] Anderson JC, Voigt CA, Arkin AP (2007). Environmental signal integration by a modular AND gate. Mol Syst Biol.

[19] Wang B, Kitney RI, Joly N, Buck M (2011). Engineering modular and orthogonal genetic logic gates for robust digital-like synthetic biology. Nat Commun.

[20] Moon TS, Lou C, Tamsir A, Stanton BC, Voigt CA (2012). Genetic programs constructed from layered logic gates in single cells. Nature.

[21] Ellis T, Wang X, Collins JJ (2009). Diversity-based, model-guided construction of synthetic gene networks with predicted functions. Nat Biotechnol.

[22] Kosuri S, Goodman DB, Cambray G, Mutalik VK, Gao Y, Arkin AP (2013). Composability of regulatory sequences controlling transcription and translation in *Escherichia Coli*. PNAS.

[23] Goodman DB, Church GM, Kosuri S (2013). Causes and effects of N-terminal codon bias in bacterial genes. Science.

[24] Davis JH, Rubin AJ, Sauer RT (2011). Design, construction and characterization of a set of insulated bacterial promoters. Nucleic Acids Res.

[25] Carr SB, Beal J, Densmore DM (2017). Reducing DNA context dependence in bacterial promoters. PLoS One.

[26] Mutalik VK, Guimaraes JC, Cambray G, Lam C, Christoffersen MJ, Mai QA (2013). Precise and reliable gene expression via standard transcription and translation initiation elements. Nat Methods.

[27] Mishra D, Rivera PM, Lin A, Del Vecchio D, Weiss R. A load driver device for engineering modularity in biological networks. Nat Biotechnol. 2014;32:1268–75. 10.1038/nbt.3044 PMC4262674 25419739

[28] Lou C, Stanton B, Chen YJ, Munsky B, Voigt CA (2012). Ribozyme-based insulator parts buffer synthetic circuits from genetic context. Nat Biotechnol.

[29] Mutalik VK, Guimaraes JC, Cambray G, Mai QA, Christoffersen MJ, Martin L (2013). Quantitative estimation of activity and quality for collections of functional genetic elements. Nat Methods.

[30] Nielsen AA, Der BS, Shin J, Vaidyanathan P, Paralanov V, Strychalski EA, et al. Genetic circuit design automation. Science. 2016; 352:aac7341. 10.1126/science.aac7341 27034378

[31] Borkowski O, Ceroni F, Stan GB, Ellis T (2016). Overloaded and stressed: whole-cell considerations for bacterial synthetic biology. Curr Opin Microbiol.

[32] Shachrai I, Zaslaver A, Alon U, Dekel E (2010). Cost of unneeded proteins in E. Coli is reduced after several generations in exponential growth. Mol Cell.

[33] Pasini M, Fernandez-Castane A, Jaramillo A, de Mas C, Caminal G, Ferrer P (2016). Using promoter libraries to reduce metabolic burden due to plasmid-encoded proteins in recombinant Escherichia Coli. Nat Biotechnol.

[34] Lo TM, Chng SH, Teo WS, Cho HS, Chang MW (2016). A two-layer gene circuit for decoupling cell growth from metabolite production. Cell Syst.

[35] Ceroni F, Algar R, Stan GB, Ellis T (2015). Quantifying cellular capacity identifies gene expression designs with reduced burden. Nat Methods.

[36] Dragosits M, Nicklas D, Tagkopoulos I. A synthetic biology approach to self-regulatory recombinant protein production in *Escherichia Coli*. J Biol Eng. 2012;6:2. 10.1186/1754-1611-6-2 PMC3384244 22463687

[37] Qian Y, Del Vecchio D. Effective interaction graphs arising from resource limitations in gene networks. Proc American Control Conference. 2015:4417–23.

[38] Gyorgy A, Jimenez JI, Yazbek J, Huang HH, Chung H, Weiss R, Del Vecchio D (2015). Isocost lines describe the cellular economy of genetic circuits. Biophys J.

[39] Carbonell-Ballestero M, Garcia-Ramallo E, Montanez R, Rodriguez-Caso C, Macia J (2016). Dealing with the genetic load in bacterial synthetic biology circuits: convergences with the Ohm’s law. Nucleic Acids Res.

[40] Algar RJR, Ellis T, Stan GB. Modelling essential interactions between synthetic genes and their chassis cell. Proc. 53rd IEEE Conference on Decision and Control. 2014; doi: 10.1109/CDC.2014.7040239.

[41] Weisse AY, Oyarzun DA, Danos V, Swain PS (2015). Mechanistic links between cellular trade-offs, gene expression, and growth. PNAS.

[42] Ang J, Harris E, Hussey BJ, Kil R, McMillen DR (2013). Tuning response curves for synthetic biology. ACS Synth Biol.

[43] Chandra FA, Del Vecchio D. The effects of ribosome autocatalysis and negative feedback in resource competition. bioRxiv. 2016; doi: 10.1101/042127

[44] Qian Y, Huang HH, Jimenez JI, Del Vecchio D. Resource competition shapes the response of genetic circuits. ACS Synth Biol. 2017; 10.1021/acssynbio.6b00361. 10.1021/acssynbio.6b00361 28350160

[45] Hooshangi S, Thiberge S, Weiss R (2005). Ultrasensitivity and noise propagation in a synthetic transcriptional cascade. PNAS.

[46] Shetty RP, Endy D, Knight TF (2008). Engineering BioBrick vectors from BioBrick parts. J Biol Eng.

[47] Elowitz MB, Leibler S. A Synthetic oscillatory network of transcriptional regulators. Nature. 2000;403:335–8. 10.1038/35002125 10659856

[48] Fuqua WC, Winans SC, Greenberg EP (1994). Quorum sensing in bacteria: the LuxR-LuxI family of cell density-responsive transcriptional regulators. J Bacteriol.

[49] Zucca S, Pasotti L, Politi N, Casanova M, Mazzini G, Cusella De Angelis MG, Magni P (2015). Multi-faceted characterization of a novel LuxR-repressible promoter library for Escherichia Coli. PLoS One.

[50] Lutz R, Bujard H (1997). Independent and tight regulation of transcriptional units in Escherichia Coli via the LacR/O, the TetR/O and AraC/I1-I2 regulatory elements. Nucleic Acids Res.

[51] Andersen JB, Sternberg C, Poulsen LK, Bjorn SP, Givskov M, Molin S (1998). New unstable variants of green fluorescent protein for studies of transient gene expression in bacteria. Appl Environ Microbiol.

[52] Zucca S, Pasotti L, Mazzini G, Cusella De Angelis MG, Magni P. Characterization of an inducible promoter in different DNA copy number conditions. BMC Bioinformatics. 2012;13(Suppl 4):S11. 10.1186/1471-2105-13-S4-S11 PMC3314568 22536957

[53] Carbonell-Ballestero M, Duran-Nebreda S, Montanez R, Sole R, Macia J, Rodriguez-Caso C. A bottom-up characterization of transfer functions for synthetic biology designs: lessons from enzymology. Nucleic Acids Res. 2014;42:14060–9. 10.1093/nar/gku964 PMC4267673 25404136

[54] Pasotti L, Quattrocelli M, Galli D, Cusella de Angelis MG, Magni P (2011). Multiplexing and demultiplexing logic functions for computing signal processing tasks in synthetic biology. Biotechnol J.

[55] Canton B, Labno A, Endy D (2008). Refinement and standardization of synthetic biological parts and devices. Nat Biotechnol.

[56] Pedraza JM, van Oudenaarden A (2005). Noise propagation in gene networks. Science.

[57] Politi N, Pasotti L, Zucca S, Magni P. Modelling the effects of cell-to-cell variability on the output of interconnected gene networks in bacterial populations. BMC Syst Biol. 2015; 9 Suppl 3:S6. 10.1186/1752-0509-9-S3-S6 PMC4464218 26050995

[58] Sleight SC, Bartley BA, Lieviant JA, Sauro HM (2010). Designing and engineering evolutionary robust genetic circuits. J Biol Eng.

[59] Cookson NA, Mather WH, Danino T, Mondragon-Palomino O, Williams RJ, Tsimring LS, Hasty J (2011). Queueing up for enzymatic processing: correlated signaling through coupled degradation. Mol Syst Biol.

[60] Knight TF (2003). Idempotent vector design for standard assembly of biobricks.

[61] MIT: Registry of Standard Biological Parts. http://partsregistry.org.

[62] Politi N, Pasotti L, Zucca S, Casanova M, Micoli G, Cusella De Angelis MG, Magni P (2014). Half-life measurements of chemical inducers for recombinant gene expression. J Biol Eng.

[63] Massaiu I, Pasotti L, Casanova M, Politi N, Zucca S, Cusella De Angelis MG, Magni P (2015). Quantification of the gene silencing performances of rationally designed synthetic small RNAs. Syst Synth Biol.

[64] Kwok R (2010). Five hard truths for synthetic biology. Nature.

[65] Purcell O, Grierson CS, Di Bernardo M, Savery NJ (2012). Temperature dependence of ssrA-tag mediated protein degradation. J Biol Eng.

