# Supplementary material for: Re-using biological devices: a model-aided analysis of interconnected transcriptional cascades designed from the bottom-up
Source: J Biol Eng. 2017 Dec 14;11:50. doi: 10.1186/s13036-017-0090-3 (PMC5729246; doi:10.1186/s13036-017-0090-3)
Supplement: Additional file 1: — Supplementary Notes. Supplementary analysis of circuits data. Supplementary Figures. Figure S1. RFP output data for the cascade circuits tested in this work (training and test set) as a function of HSL concentration. Figure S2. Growth rate data for the cascade circuits tested in this work (training and test set) as a function of HSL concentration. Figure S3. GFP output data for the cascade circuits with Monitor cassette tested in this work (training and test set) as a function of HSL concentration. Figure S4. Growth rate (A) and RFP (B) comparison plots between strains without and with Monitor cassette at all the tested HSL concentrations for all the circuits (training and test set). Figure S5. Correlation between GFP and growth rate in all the strains (training and test set) with the Monitor cassette at all the tested HSL concentrations. Figure S6. Correlation between GFP and growth rate for each strain with the Monitor cassette at all the tested HSL concentrations. Figure S7. Correlation between GFP and RFP for each strain with the Monitor cassette at all the tested HSL concentrations. Figure S8. NBM fitting of the measured HSL-dependent output in all the training set circuits without Monitor cassette. Figure S9. NBM fitting of the two NOT gate characteristics as a function of the predicted per cell concentration of the TetR or LacI repressor. Figure S10. Univariate sensitivity analysis of the NBM by applying a variation on the δ parameter of the Hill functions. Figure S11. Univariate sensitivity analysis of the NBM by applying a variation on the α parameter of the Hill functions. Figure S12. Univariate sensitivity analysis of the NBM by applying a variation on the K parameter of the Hill functions. Figure S13. Univariate sensitivity analysis of the NBM by applying a variation on the η parameter of the Hill functions. Figure S14. Evolutionary stability of the X1TLr and XrepTLr circuits. Figure S15. Simulation of X1TLr and XrepTLr with the NBM for different values [file 13036_2017_90_MOESM1_ESM.pdf]

Supplementary Notes

Supplementary analysis of circuits data

The maximum output value and switch point of the input devices with P_{lux} and P_{luxRep} (X_1r, X_2r, X_3r and X_{rep}r) is consistent with previous studies where similar circuits were characterized [15, 49, 52, 53, 54, 55]. Among these studies, a circuit analogous to X_3r, in the same plasmid, gave a maximum activity value of 8 RPU [52] and the half-maximum value was at ~2 nM HSL [52, 55]. A different study with a circuit identical to X_2r, however, reported a lower maximum activity (4 RPU) and a much higher switch point (>700 nM) than observed here [15]. All the mentioned studies were carried out in different *E. coli* strains, which might explain the observed quantitative differences. In particular, in the latter case [15], the strain constitutively over-expressed LacI, resulting in a low LuxR production which can affect the static transfer function by lowering its maximum value and increasing the switch point [53]. The characterization of X_1r was also available from a previous study [54] in TOP10, but in a high-copy vector and in non-standard units, yielding a switch point (~1 nM of HSL) comparable to the one in the present work. Analogously, P_{luxRep} showed a consistent behaviour with the previously reported characterization [49].

Among the 2-block cascades, the TetR-based NOT gate characterization showed a higher maximum activity than previously reported in a different strain, but the input level, in terms of RPU, that causes a half-maximum response was highly consistent [15]. The LacI-based NOT gate was not characterized previously in a circuit with this RBS. As expected, the maximum value of P_{LlacO1} was much lower than previously reported in the same strain (~6 RPU) or a different one (~2.5 RPU) [15], since it was always characterized in presence of the gratuitous inducer isopropyl β-D-1-thiogalactopyranoside (IPTG), which binds the endogenously expressed LacI repressor of P_{LlacO1}. In the strain used in this study, LacI is constitutively expressed at low level, resulting in a relatively low output for P_{LlacO1} (~0.5 RPU).

Supplementary Figures

Figure S1. RFP output data for the cascade circuits tested in this work (training and test set) as a function of HSL concentration. Circles represent the average measured values, while error bars represent the 95% confidence intervals of the mean. Magenta and blue colours correspond to the RFP output of circuits without (*r* suffix) and with (*rg* suffix) Monitor cassette, respectively.

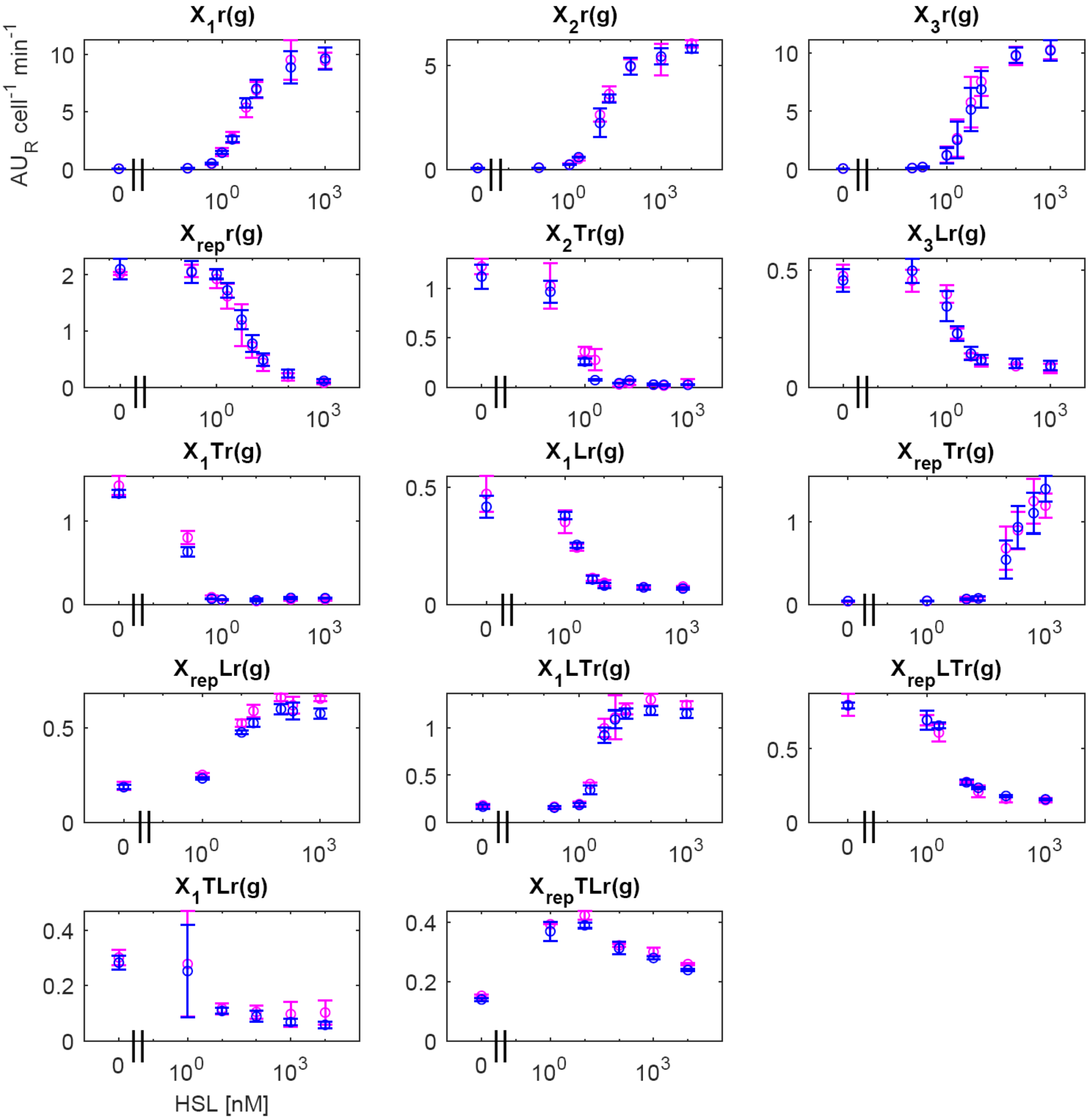

Figure S2. Growth rate data for the cascade circuits tested in this work (training and test set) as a function of HSL concentration. Circles represent the average measured values, while error bars represent the 95% confidence intervals of the mean. Magenta and blue colours correspond to the growth rate of circuits without (*r* suffix) and with (*rg* suffix) Monitor cassette, respectively.

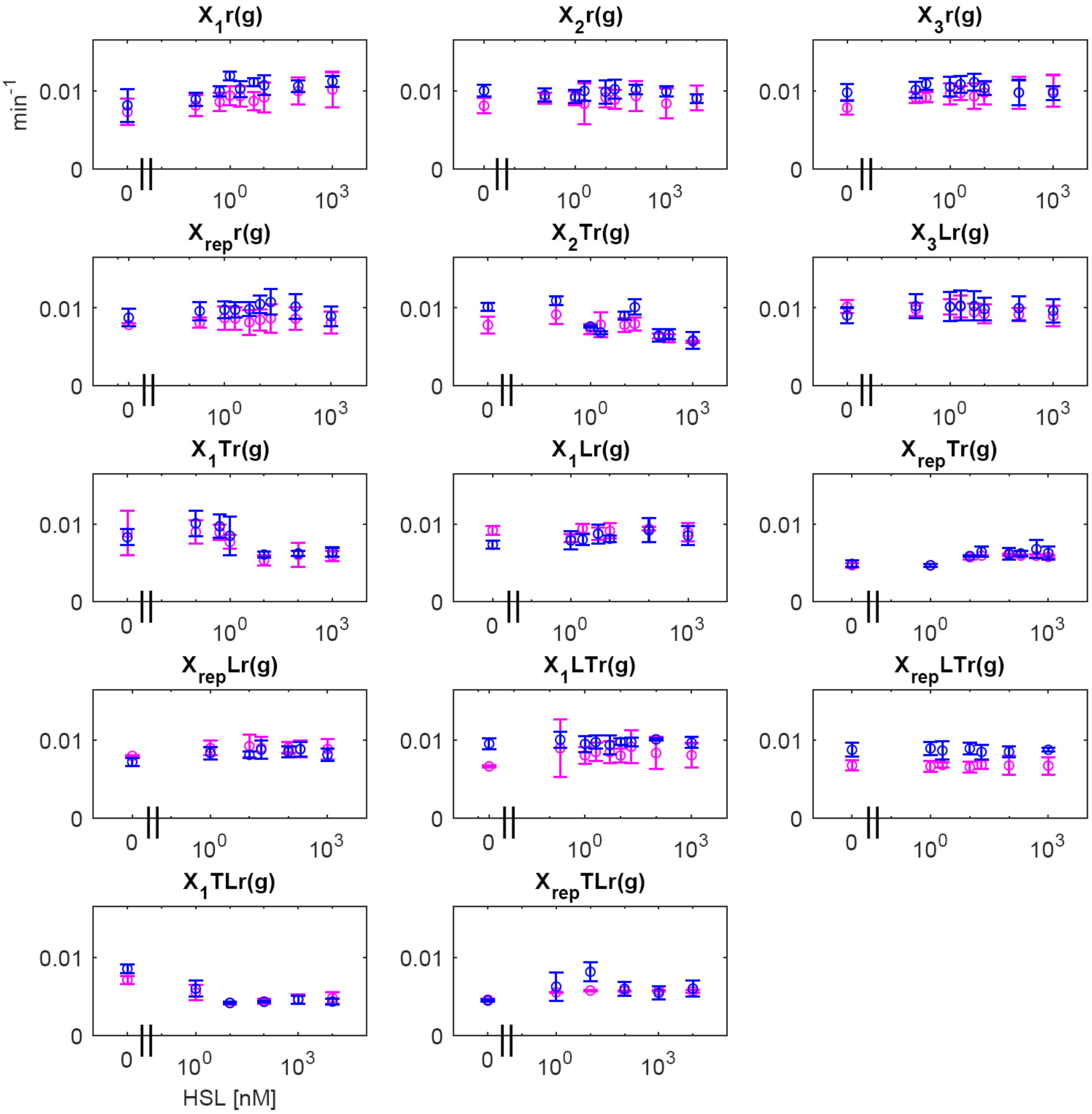

Figure S3. GFP output data for the cascade circuits with Monitor cassette tested in this work (training and test set) as a function of HSL concentration. Circles represent the average measured values, while error bars represent the 95% confidence intervals of the mean.

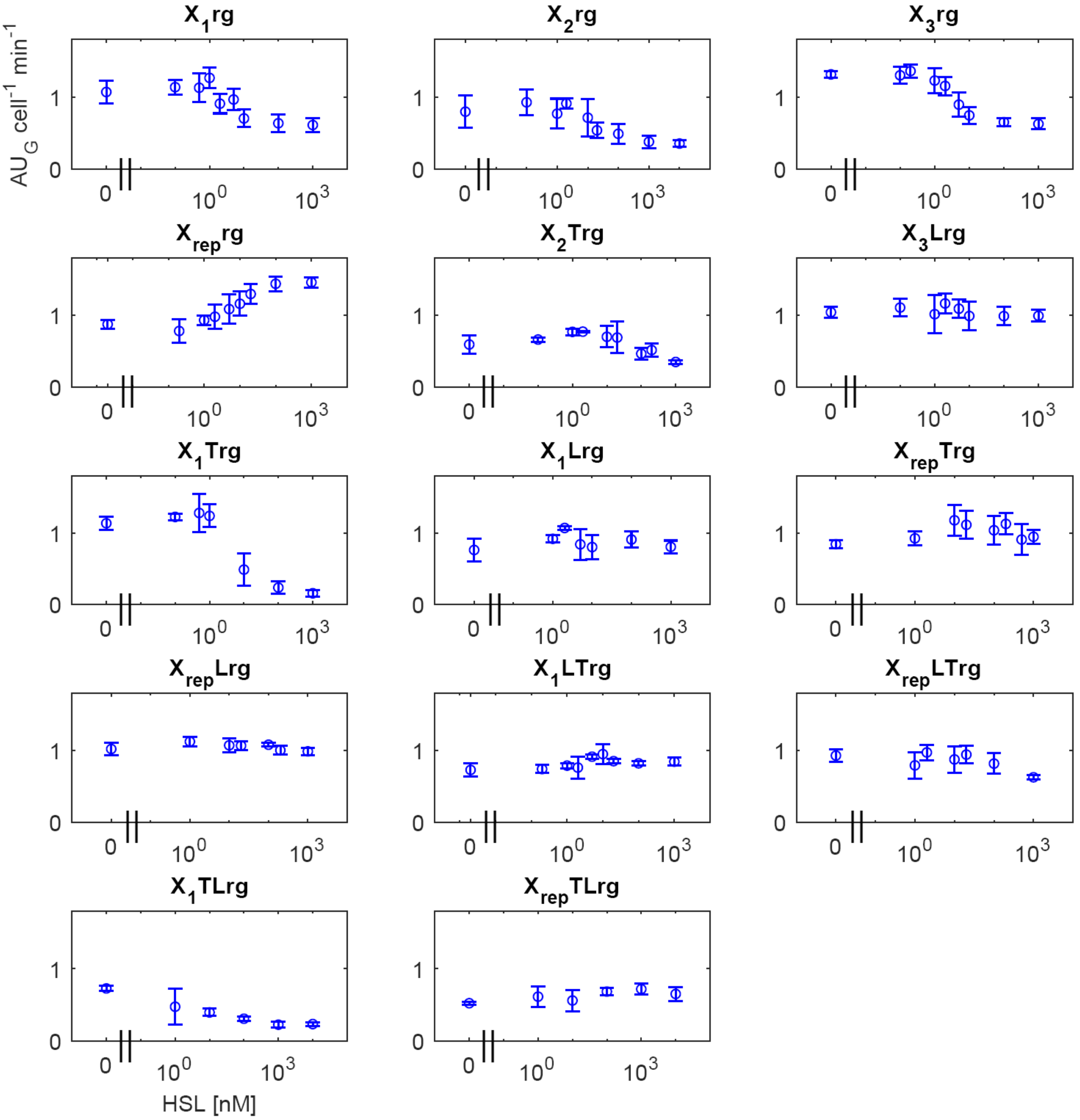

Figure S4. Growth rate (A) and RFP (B) comparison plots between strains without and with Monitor cassette at all the tested HSL concentrations for all the circuits (training and test set). Circles represent the average values, while solid line represents the bisector line.

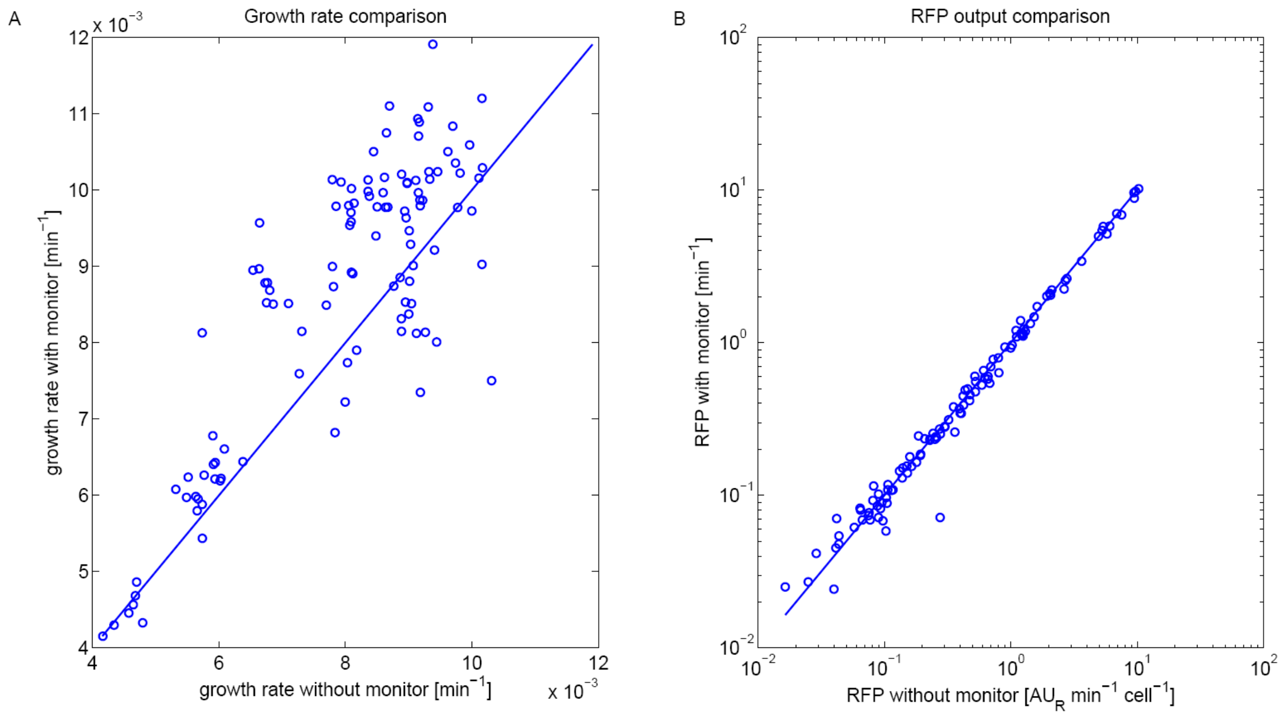

Figure S5. Correlation between GFP and growth rate in all the strains (training and test set) with the Monitor cassette at all the tested HSL concentrations. Circles represent the individual values in all the biological replicates, while solid line represents the regression line.

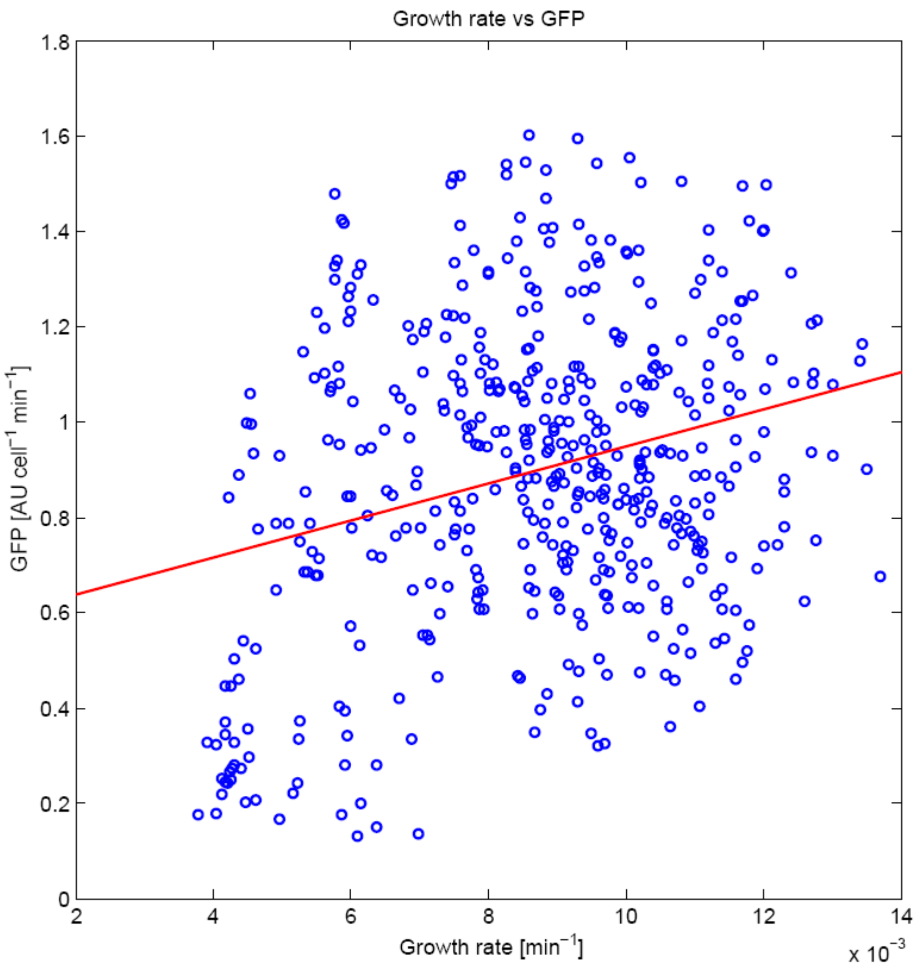

Figure S6. Correlation between GFP and growth rate for each strain with the Monitor cassette at all the tested HSL concentrations. Circles represent the individually measured values in all the biological replicates.

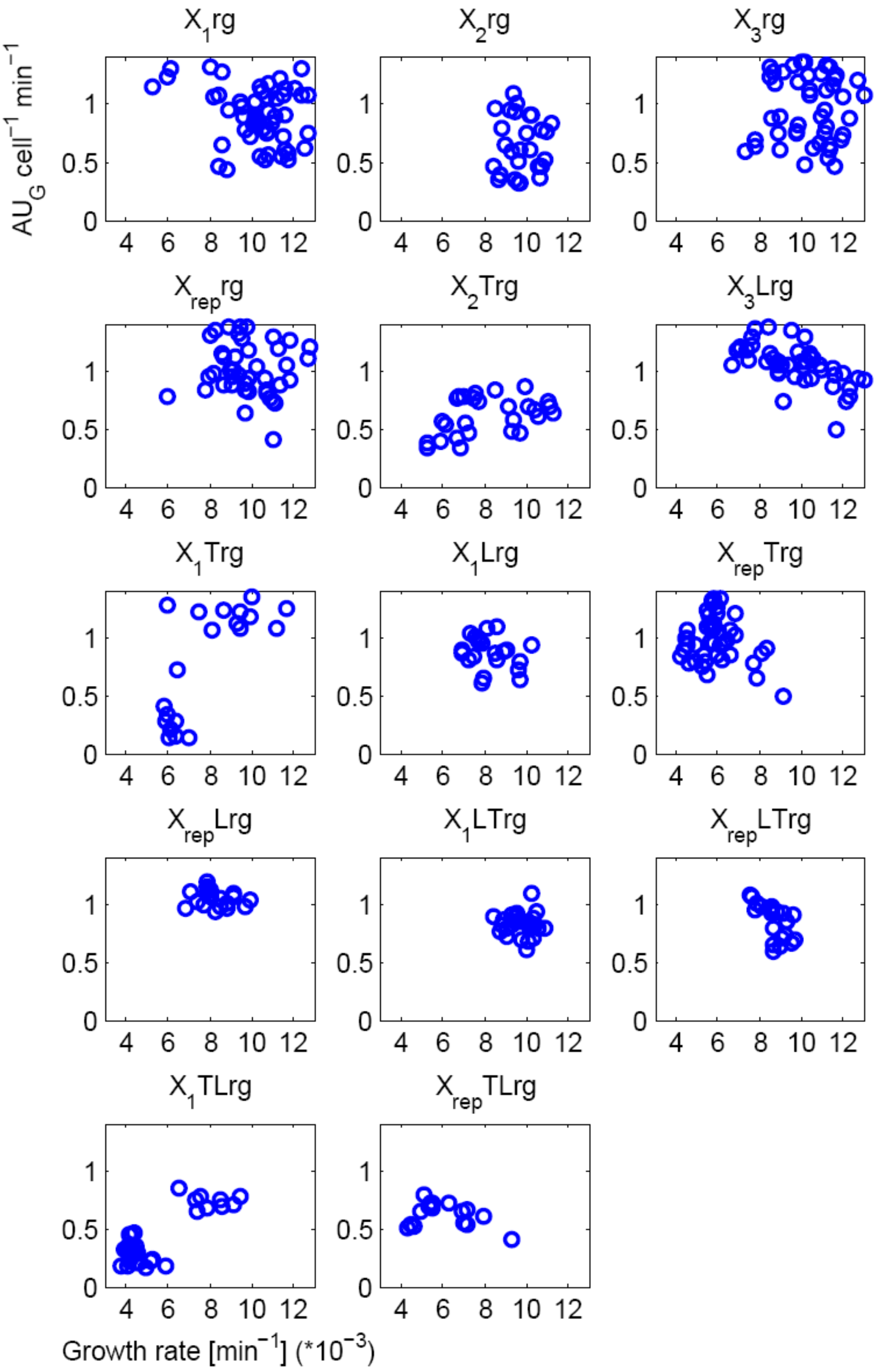

Figure S7. Correlation between GFP and RFP for each strain with the Monitor cassette at all the tested HSL concentrations. Circles represent the individually measured values in all the biological replicates.

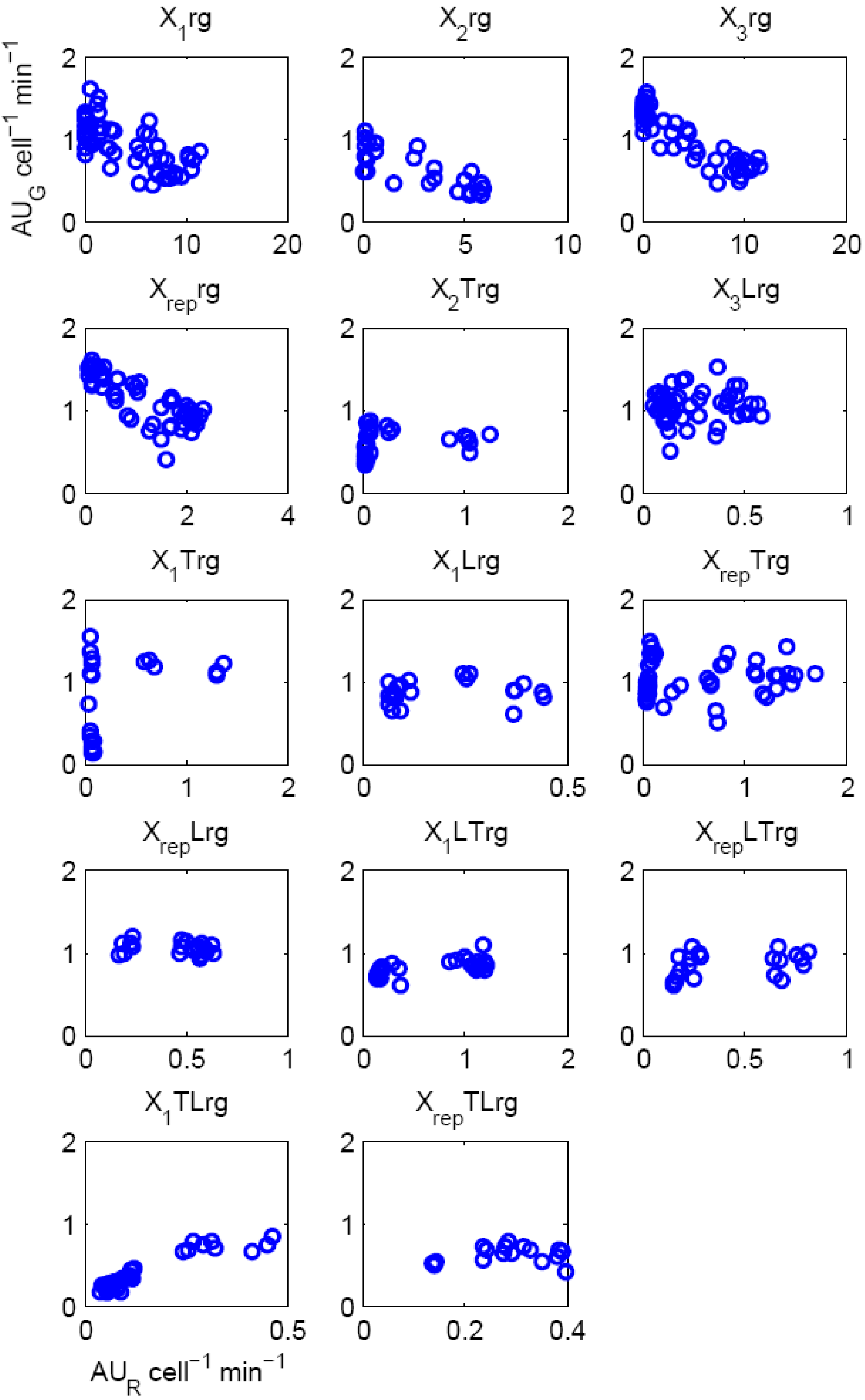

Figure S8. NBM fitting of the measured HSL-dependent output in all the training set circuits without Monitor cassette. Circles represent the average measured value and error bars represent the 95% confidence intervals of the mean. Solid line represents the median predicted output of the model calculated via Monte Carlo simulations for each HSL concentration tested. Dashed dark red lines are the 95% confidence bands of the output distribution. Dashed light red lines are the 95% confidence bands of the output distribution calculated after multivariate sensitivity analysis.

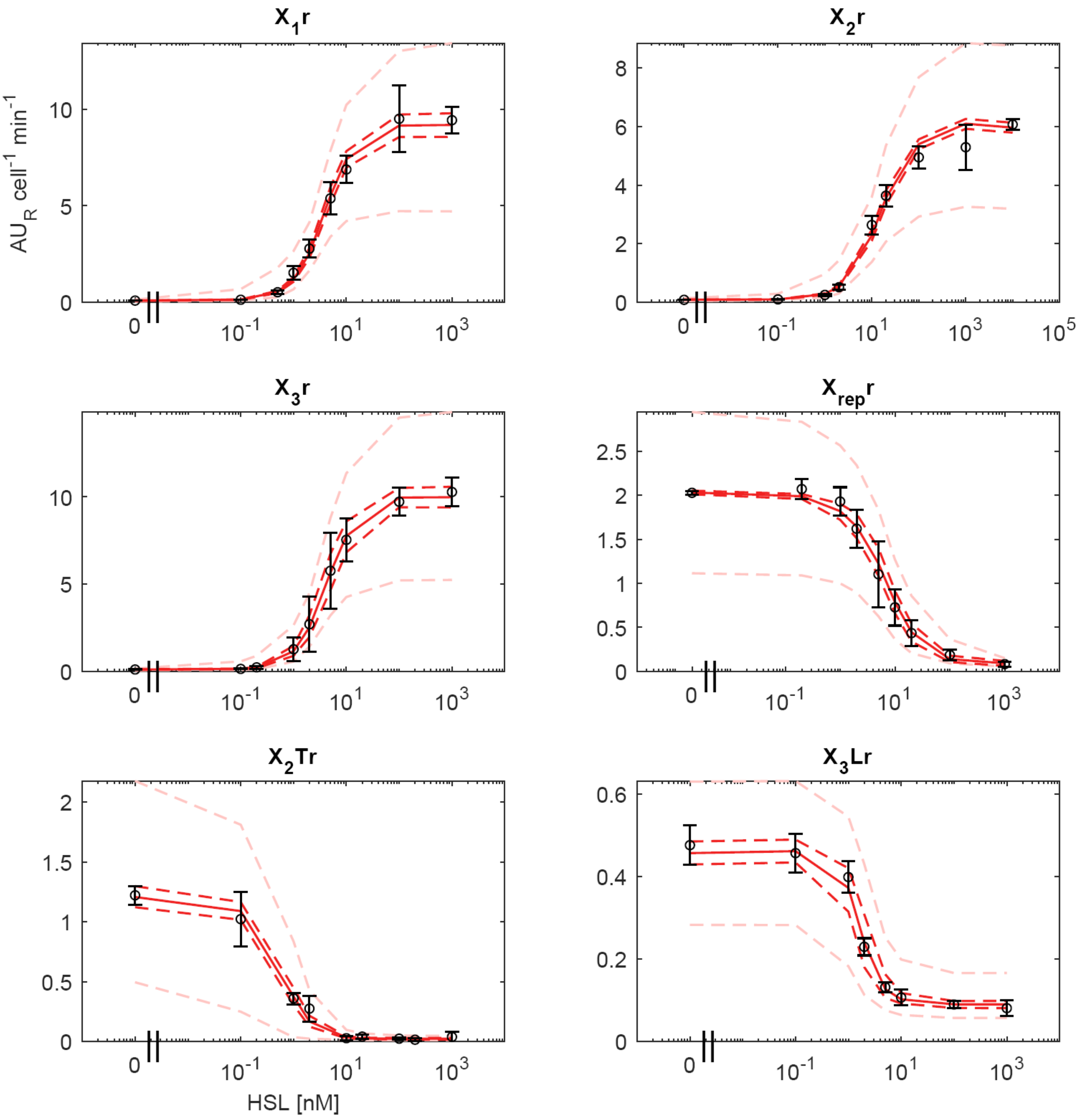

Figure S9. NBM fitting of the two NOT gate characteristics as a function of the predicted per cell concentration of the TetR or LacI repressor. Circles represent the average measured value and error bars represent the 95% confidence intervals of the mean. Solid line represents the median predicted output of the model calculated via Monte Carlo simulations for each HSL concentration tested. Dashed dark red lines are the 95% confidence bands of the output distribution. Dashed light red lines are the 95% confidence bands of the output distribution calculated after multivariate sensitivity analysis. The TetR and LacI values in the x-axes were computed as the nominal (i.e., without Monte Carlo approach) values predicted by the model. The data points showed for TetR or LacI = 0 were obtained by measuring the output of constructs similar to X_2Tr(g) and X_3Lr(g) but without their input block, and were used in the fitting procedure. This genetic context enables to measure the activity of P_{LtetO1} and P_{LlacO1} in absence of their cognate repressor, which cannot be removed in the X_2Tr(g) and X_3Lr(g) circuits due to the basic activity of promoters in the X_2 and X_3 input blocks.

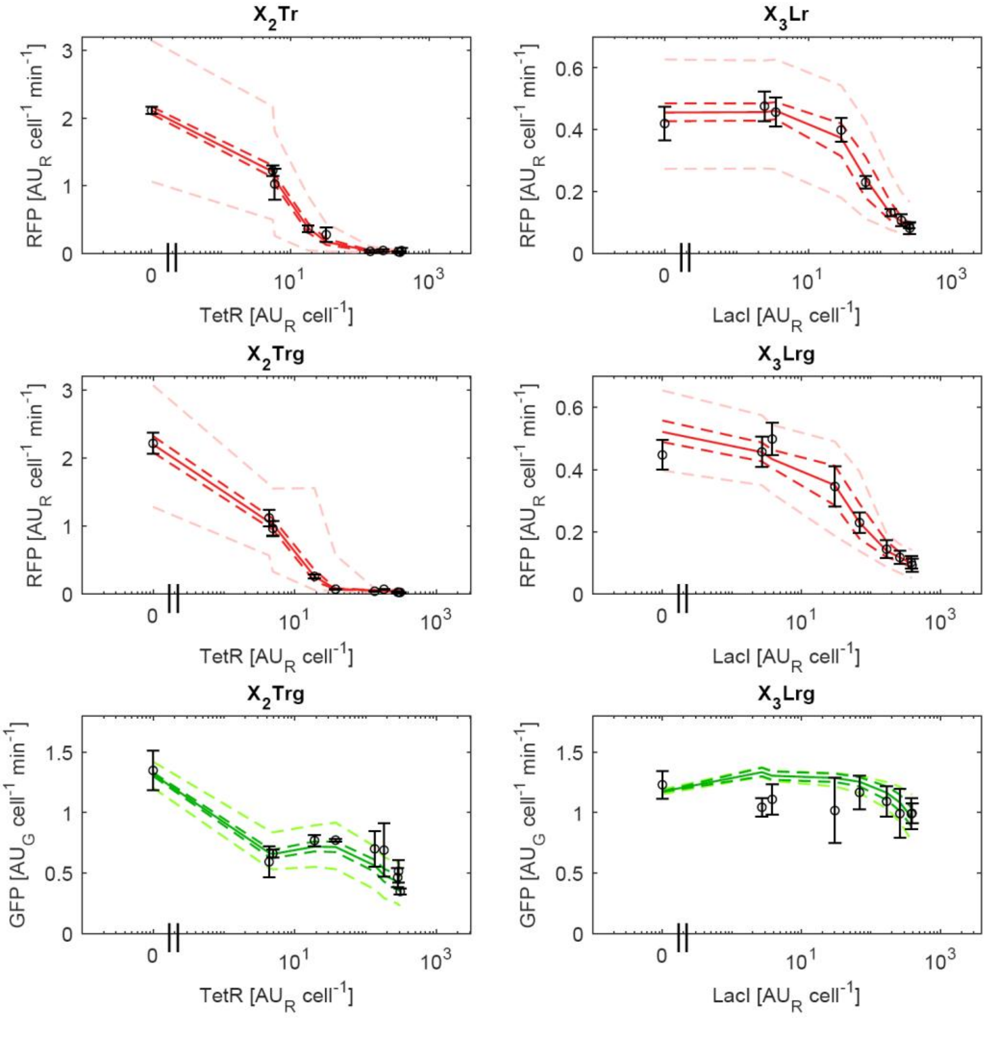

Figure S10. Univariate sensitivity analysis of the NBM by applying a variation on the δ parameter of the Hill functions. Panels show fitting (X_1r, X_2r, X_3r, X_{rep}r, X_2Tr and X_3Lr circuits) and predictions (remaining circuits) of the measured HSL-dependent output in all the training and test set circuits without Monitor cassette. Circles represent the average measured value and error bars represent the 95% confidence intervals of the mean. Solid line represents the median predicted output of the model calculated via Monte Carlo simulations for each HSL concentration tested. Dashed dark red lines are the 95% confidence bands of the output distribution. Dashed light red lines are the 95% confidence bands of the output distribution calculated after univariate sensitivity analysis.

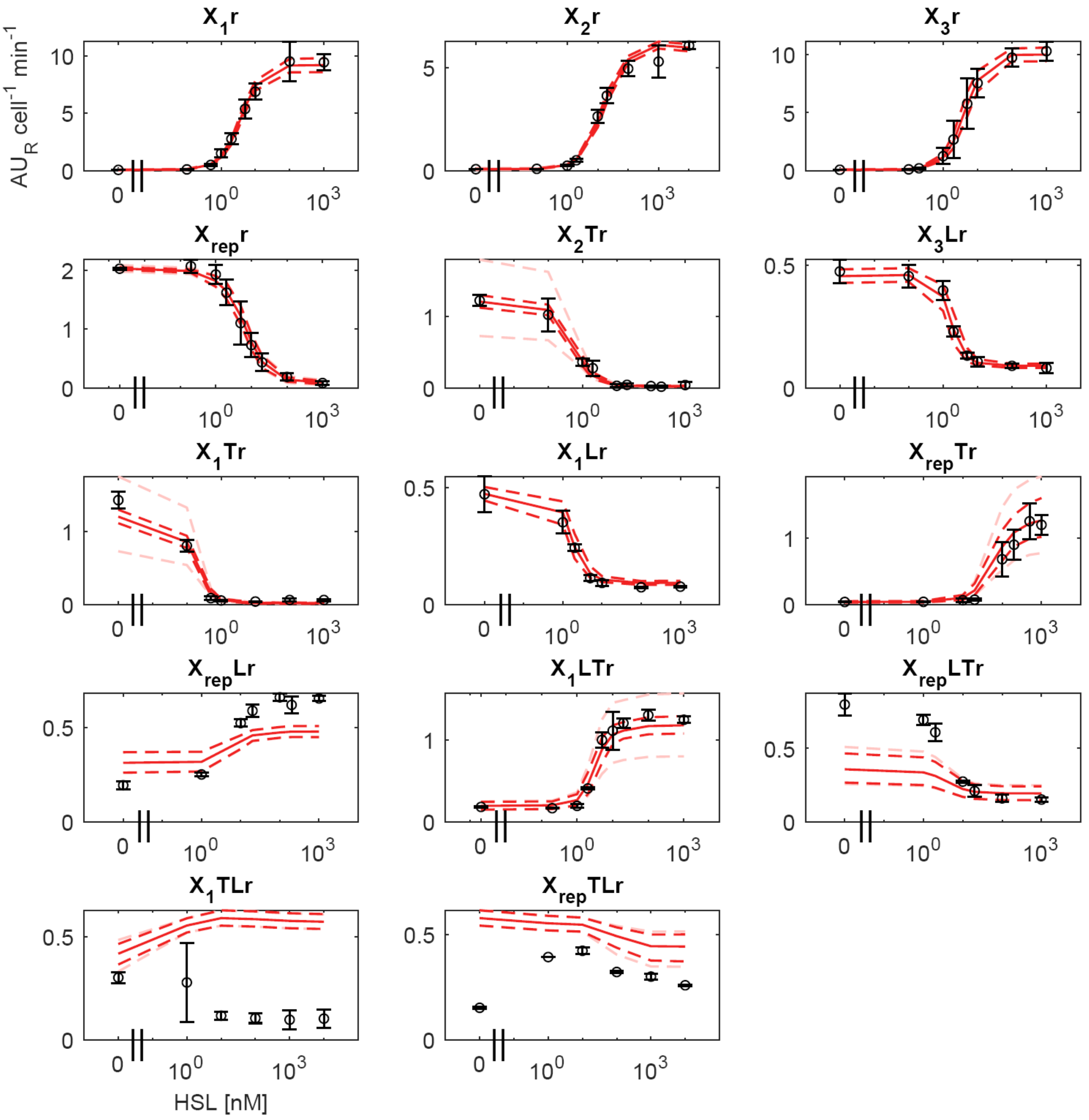

Figure S11. Univariate sensitivity analysis of the NBM by applying a variation on the α parameter of the Hill functions. Panels show fitting (X_1r, X_2r, X_3r, X_{rep}r, X_2Tr and X_3Lr circuits) and predictions (remaining circuits) of the measured HSL-dependent output in all the training and test set circuits without Monitor cassette. Circles represent the average measured value and error bars represent the 95% confidence intervals of the mean. Solid line represents the median predicted output of the model calculated via Monte Carlo simulations for each HSL concentration tested. Dashed dark red lines are the 95% confidence bands of the output distribution. Dashed light red lines are the 95% confidence bands of the output distribution calculated after univariate sensitivity analysis.

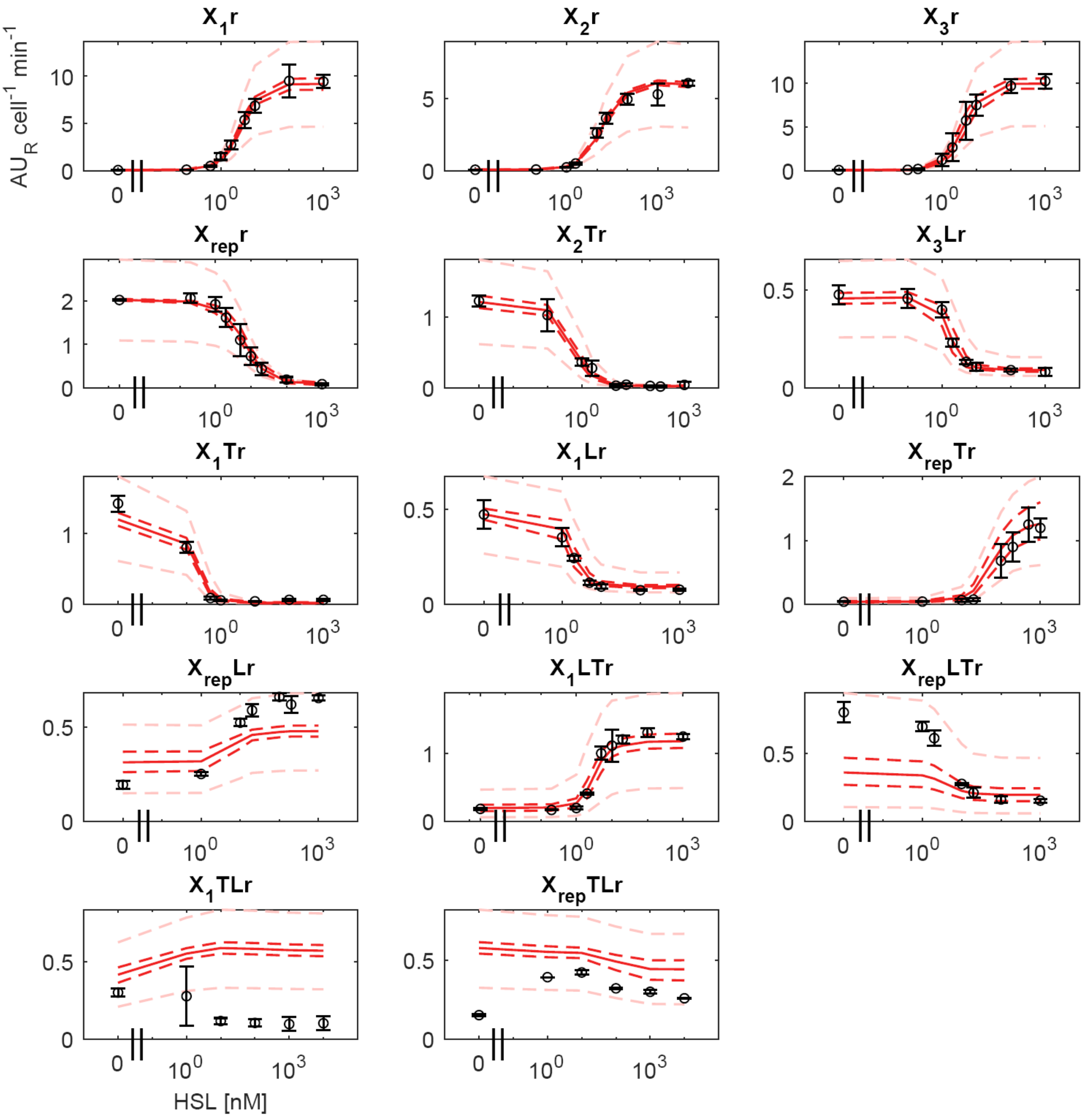

Figure S12. Univariate sensitivity analysis of the NBM by applying a variation on the K parameter of the Hill functions. Panels show fitting (X_1r, X_2r, X_3r, X_{rep}r, X_2Tr and X_3Lr circuits) and predictions (remaining circuits) of the measured HSL-dependent output in all the training and test set circuits without Monitor cassette. Circles represent the average measured value and error bars represent the 95% confidence intervals of the mean. Solid line represents the median predicted output of the model calculated via Monte Carlo simulations for each HSL concentration tested. Dashed dark red lines are the 95% confidence bands of the output distribution. Dashed light red lines are the 95% confidence bands of the output distribution calculated after univariate sensitivity analysis.

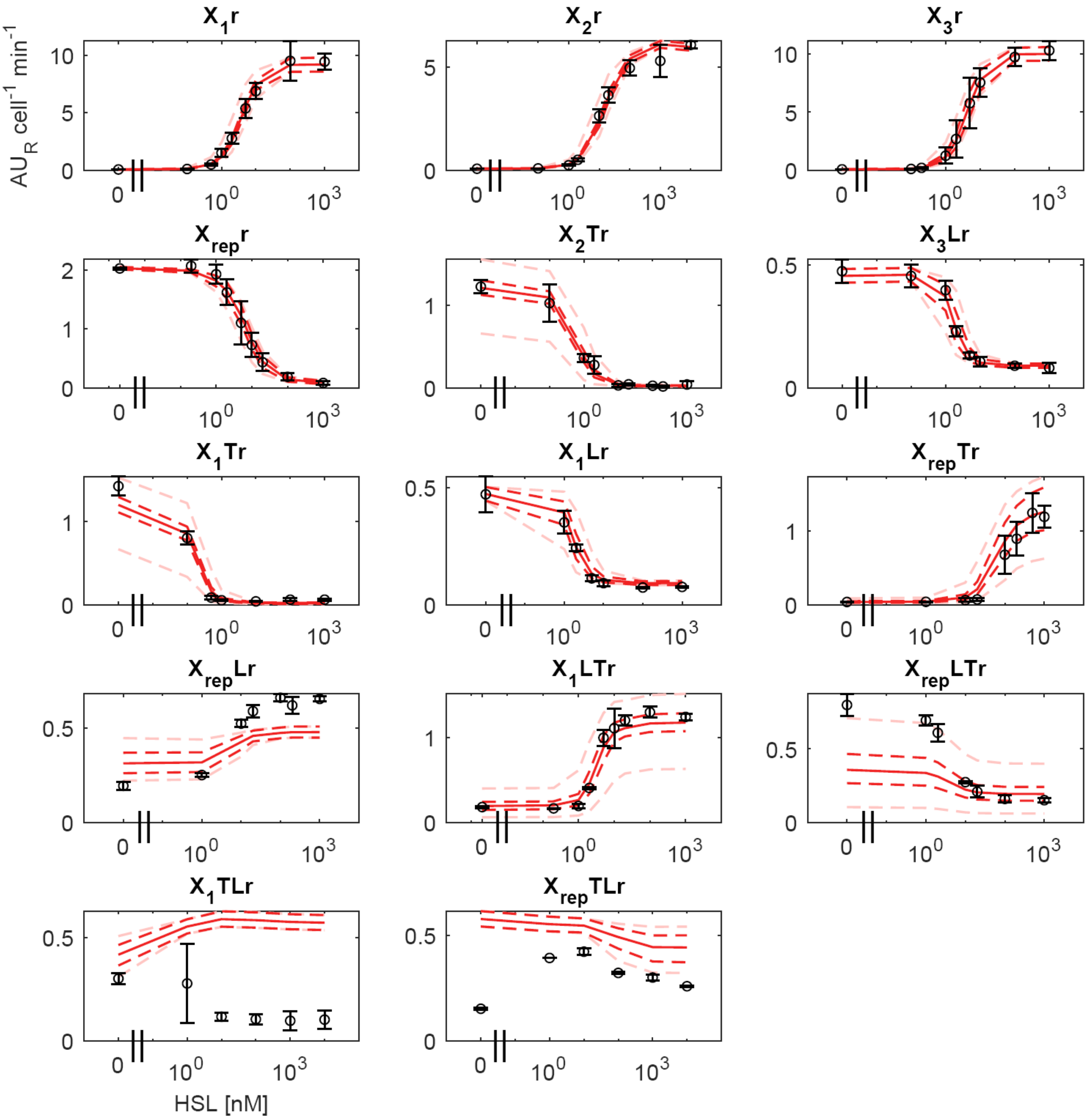

Figure S13. Univariate sensitivity analysis of the NBM by applying a variation on the η parameter of the Hill functions. Panels show fitting (X_1r, X_2r, X_3r, $X_{rep}r$, X_2Tr and X_3Lr circuits) and predictions (remaining circuits) of the measured HSL-dependent output in all the training and test set circuits without Monitor cassette. Circles represent the average measured value and error bars represent the 95% confidence intervals of the mean. Solid line represents the median predicted output of the model calculated via Monte Carlo simulations for each HSL concentration tested. Dashed dark red lines are the 95% confidence bands of the output distribution. Dashed light red lines are the 95% confidence bands of the output distribution calculated after univariate sensitivity analysis.

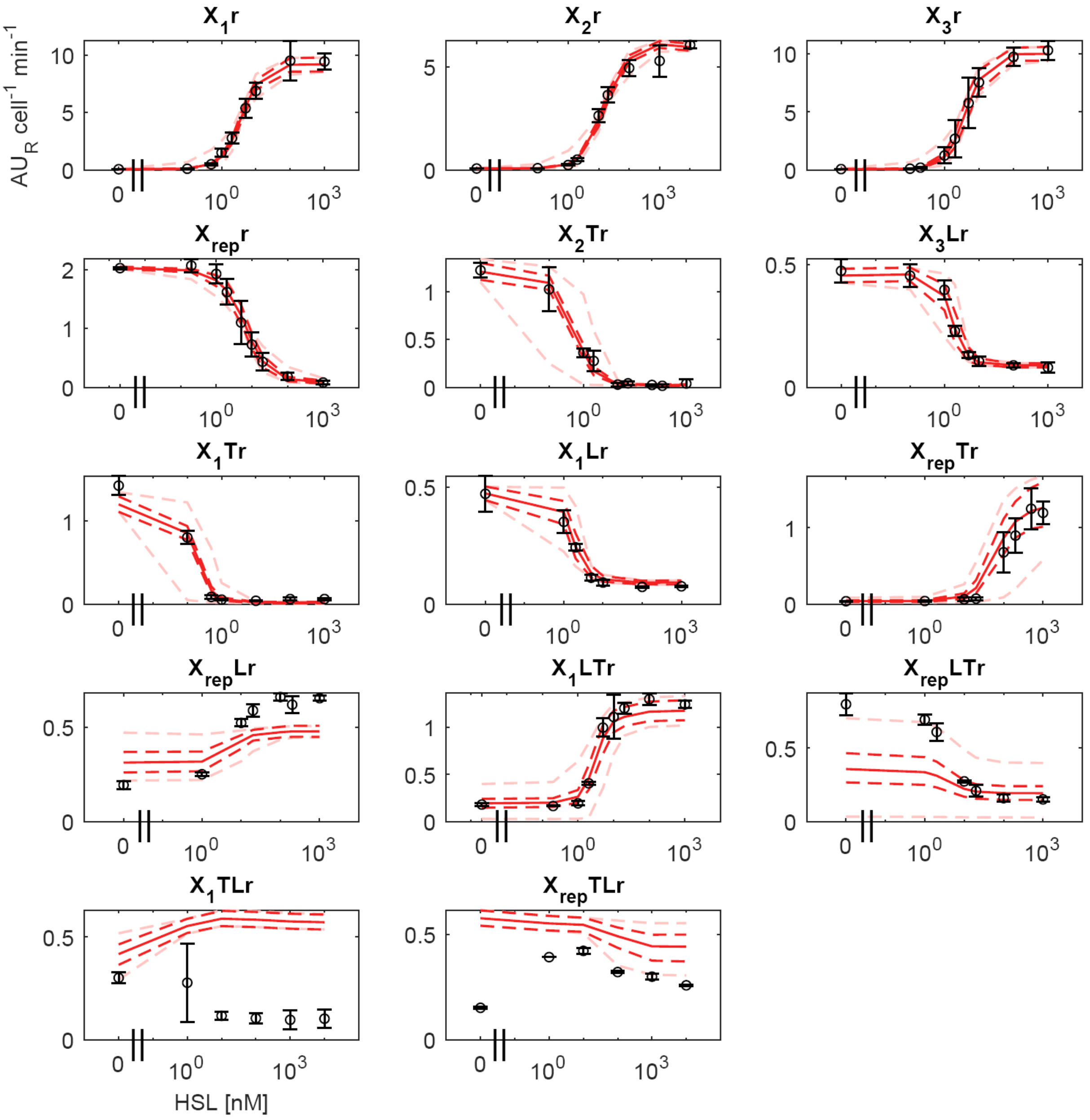

Figure S14. Evolutionary stability of the X_1TLr and X_{rep}TLr circuits. A) Phenotypic stability of strains with X_1TLr and X_{rep}TLr. Strains were tested at three different HSL concentrations (test#1), reported in the x-axis, and then re-inoculated and tested in a growth medium without HSL (X_1TLr) or with 10000 nM of HSL (X_{rep}TLr) (test#2). In this experiment, we evaluated if the strains could restore the RFP output observed at zero (X_1TLr) or full induction (X_{rep}TLr), corresponding to conditions in which the expression of TetR is repressed, after an experiment carried out at different HSL concentrations. Data points represent the mean of three biological replicates and error bars represent the 95% confidence intervals of the mean. B) Genetic stability of the two strains. Electrophoresis results (ethidium bromide staining) are shown for all the tested strains and HSL concentrations after the experiment above. A description of the GeneRuler 1Kb DNA ladder (Thermo Scientific) is also provided (adapted from the user guide of product #SM0312, Thermo Scientific). The mutation found in the second replicate of X_1TLr (previously tested with 10000 nM of HSL) is reported.

Protocol for Panel A. Cultures were tested in microplate reader as described in the Methods section, with HSL concentrations of 0, 10 and 10000 nM (X_1TLr) or 0, 1 and 10000 nM (X_{rep}TLr). At the end of the test (18-h growth in microplate reader), all the X_1TLr cultures were centrifuged, the supernatant was removed and the pellet was resuspended with 200 µl of fresh selective medium without HSL. This washing step was performed to remove HSL from the induced X_1TLr cultures. Five hundred µl of M9 were inoculated with 5 µl of the X_1TLr (washed) or X_{rep}TLr cultures in 2-ml tubes. HSL (final concentration of 10000 nM) was added to the X_{rep}TLr cultures. All the cultures were incubated overnight at 37°C, 220 rpm, and then they were tested again in the microplate reader in absence of HSL (X_1TLr) or with 10000 nM of HSL (X_{rep}TLr).

Results: all the strains showed stable behaviour, since the RFP output in test#2 is comparable to the RFP output in test#1 for HSL=0 (X_1TLr) or 10000 nM (X_{rep}TLr), suggesting that HSL-dependent RFP changes in test#1 were not due to stability mutants.

Protocol for Panel B. In parallel with the inoculation of the 500-µl cultures, 2 µl of the X_1TLr (washed) or X_{rep}TLr cultures were used to inoculate 10 ml of selective medium in 50-ml tubes. Cultures were grown overnight at 37°C, 220 rpm. Plasmid DNA was purified, digested with EcoRI-PstI and ran on 1% agarose gel. The second and first biological replicates of X_1TLr and X_{rep}TLr, respectively, were sequenced with primers C0062VF (5'-GAATGTTTAGCGTGGGCATG-3') and VR (5'-ATTACCGCCTTTGAGTGAGC-3').

Results: from electrophoresis screening, all the constructs showed the correct bands, corresponding to vector backbone (3.2 Kbp) and insert (4.1 Kbp); however, X_1TLr cultures grown in test#1 with HSL=10000 and 10 nM also showed bands of unexpected size. From sequencing results, only the X_1TLr culture grown in test#1 with 10000 nM of HSL showed DNA alterations, in a small portion of the population (according to the chromatogram), while the other sequenced plasmids did not show detectable mutations with the used primers. The observed mutation was a deletion of all the circuit after the luxR gene and before the transcriptional terminator of the RFP gene.

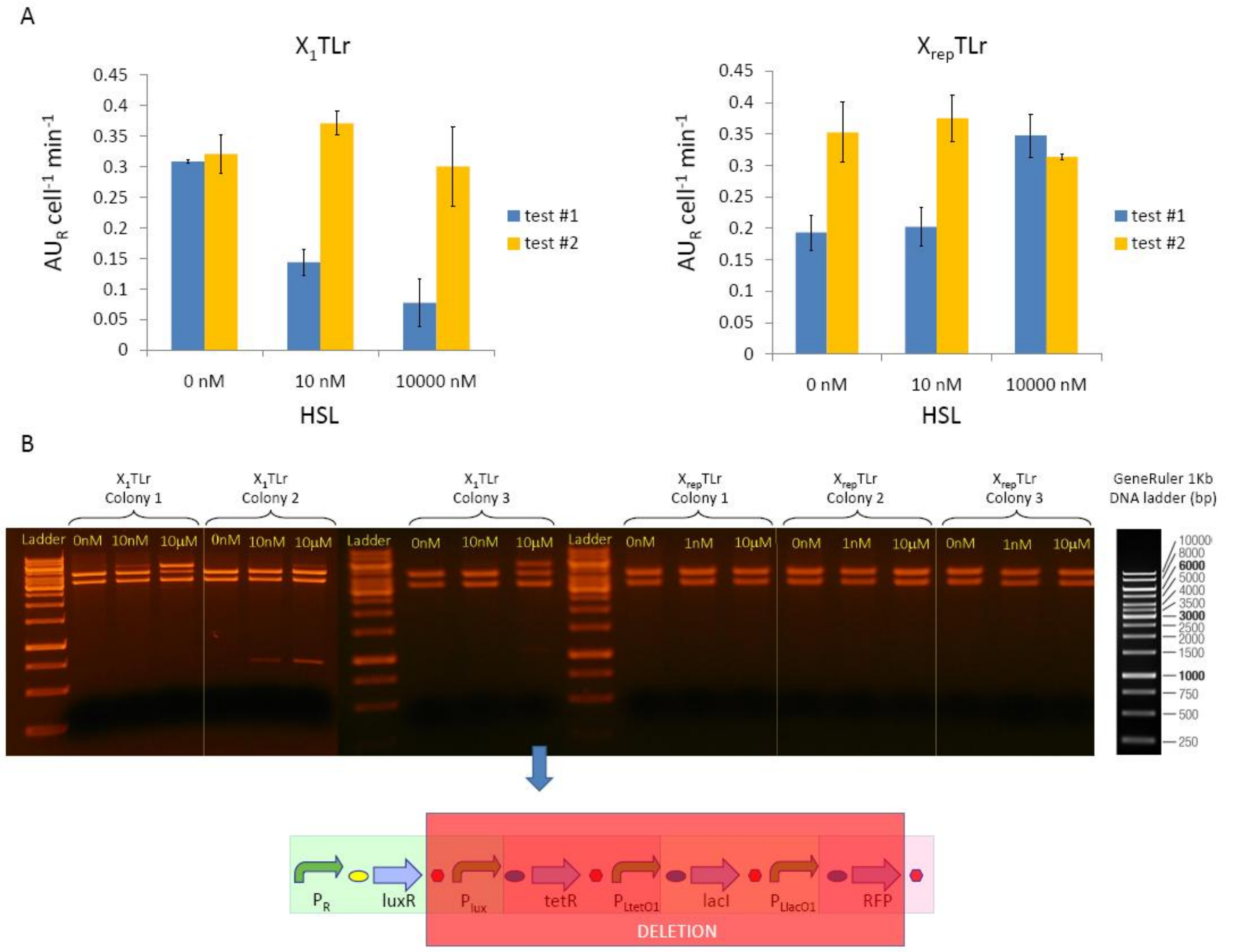

Figure S15. Simulation of X_1TLr and X_{rep}TLr with the NBM for different values of γ_{tet} and γ_{lac} parameters. Data are reported (circles and error bars represent the 95% confidence intervals of the mean) and the simulated RFP output is shown (solid line). The parameters of the NBM reported in Table 1 were used for the simulations. The γ_{tet} and γ_{lac} parameter values were set at the nominal ones (see Methods section) or they were decreased by 100-fold (marked as "low") to qualitatively evaluate the effect of enzymatic queuing, which might cause slower TetR and LacI degradation, since they share the same LVA tag.

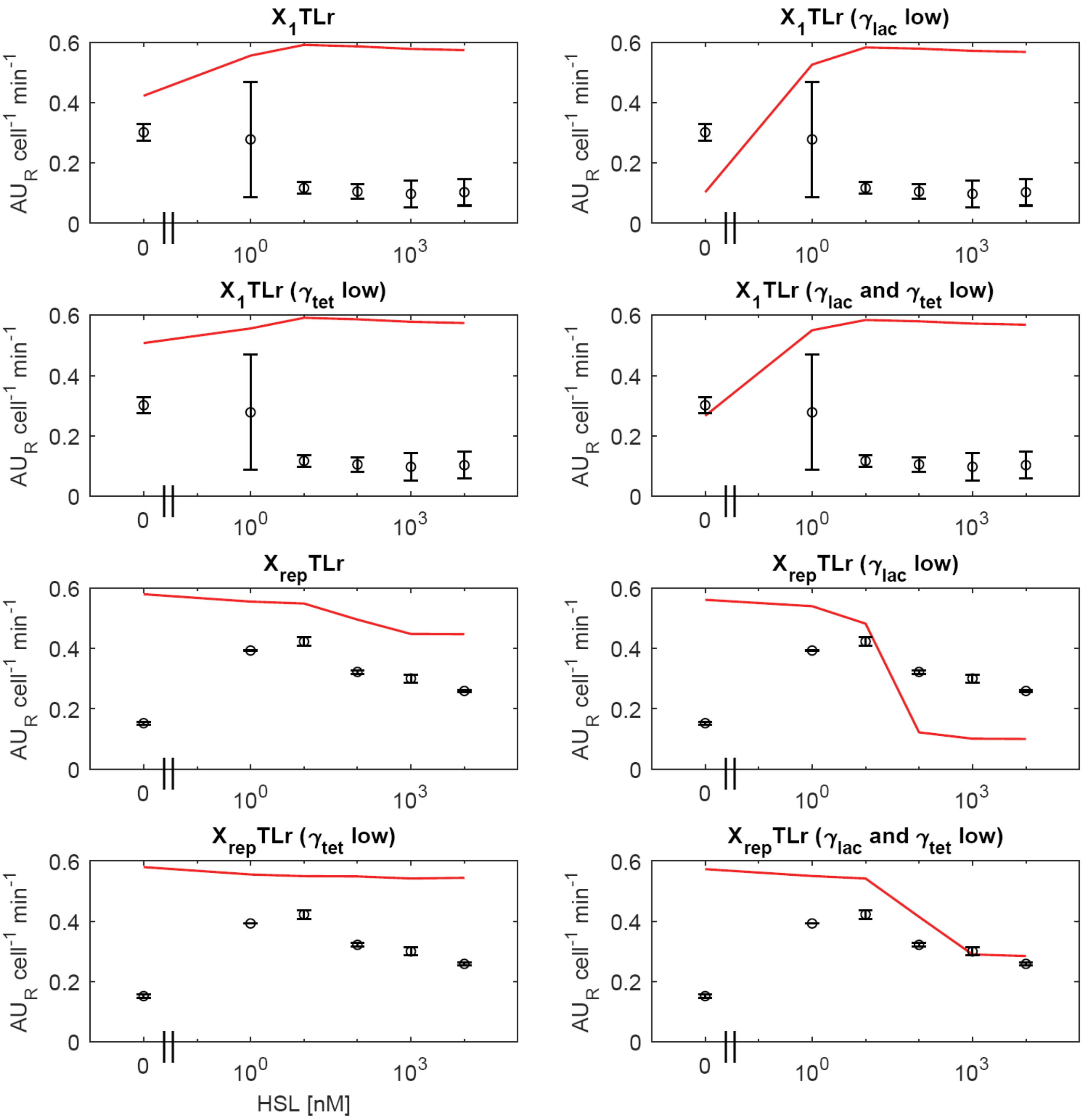

Figure S16. Fitting and prediction results for the NBM learned and simulated against RFP data of the circuits with the Monitor cassette. Panels show fitting (X_1r, X_2r, X_3r, X_{rep}r, X_2Tr and X_3Lr circuits) and predictions (remaining circuits) of the measured HSL-dependent output in all the training and test set circuits. Circles represent the average measured value and error bars represent the 95% confidence intervals of the mean. Solid line represents the median predicted output of the model calculated via Monte Carlo simulations for each HSL concentration tested. Dashed dark red lines are the 95% confidence bands of the output distribution. Dashed light red lines are the 95% confidence bands of the output distribution calculated after multivariate sensitivity analysis.

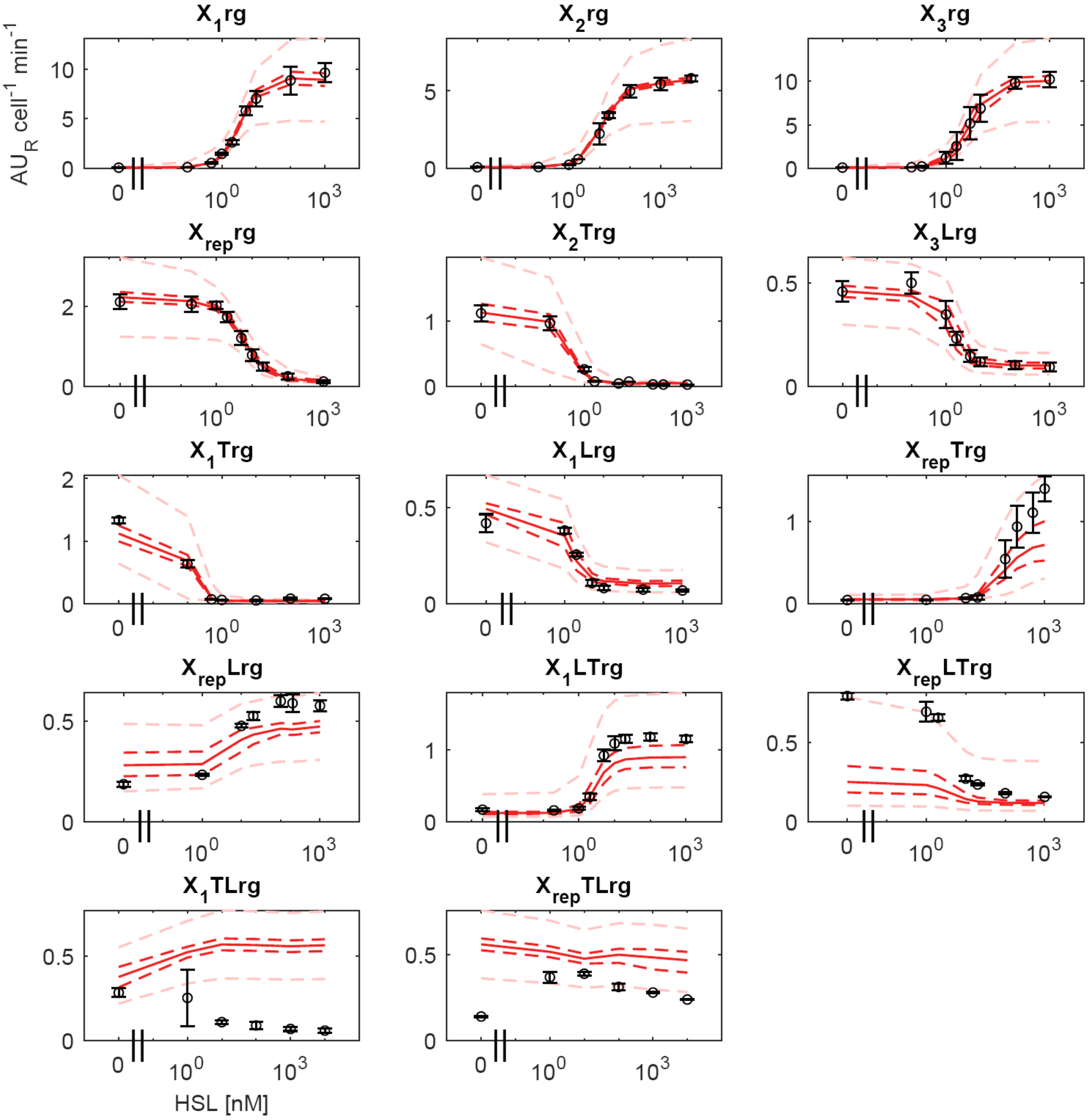

Figure S17. BM fitting of the measured HSL-dependent RFP output in all the training set circuits with Monitor cassette. Circles represent the average measured value and error bars represent the 95% confidence intervals of the mean. Solid line represents the median predicted output of the model calculated via Monte Carlo simulations for each HSL concentration tested. Dashed dark red lines are the 95% confidence bands of the output distribution. Dashed light red lines are the 95% confidence bands of the output distribution calculated after multivariate sensitivity analysis.

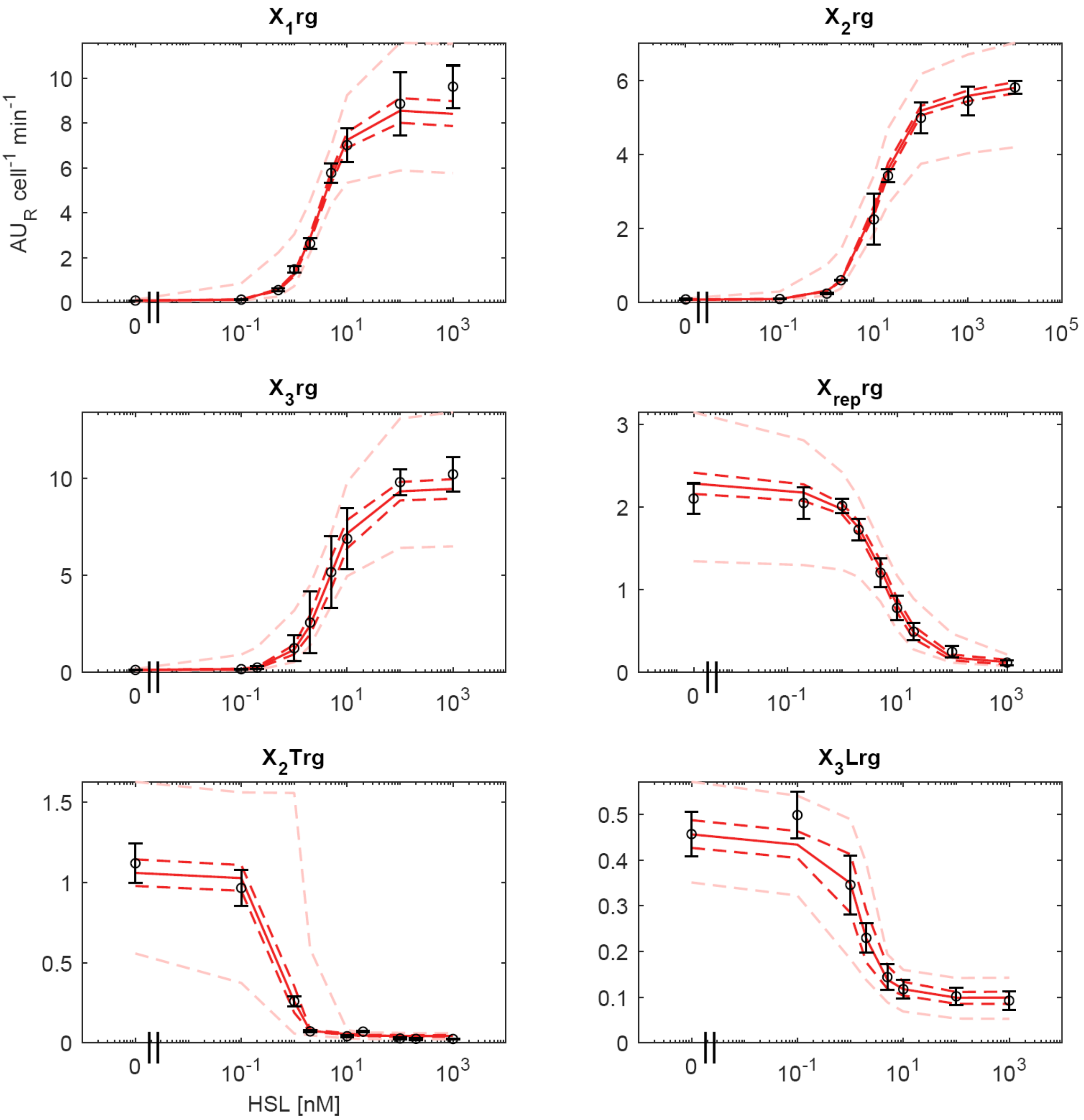

Figure S18. BM fitting of the measured HSL-dependent GFP output in all the training set circuits with Monitor cassette. Circles represent the average measured value and error bars represent the 95% confidence intervals of the mean. Solid line represents the median predicted output of the model calculated via Monte Carlo simulations for each HSL concentration tested. Dashed dark green lines are the 95% confidence bands of the output distribution. Dashed light green lines are the 95% confidence bands of the output distribution calculated after multivariate sensitivity analysis.

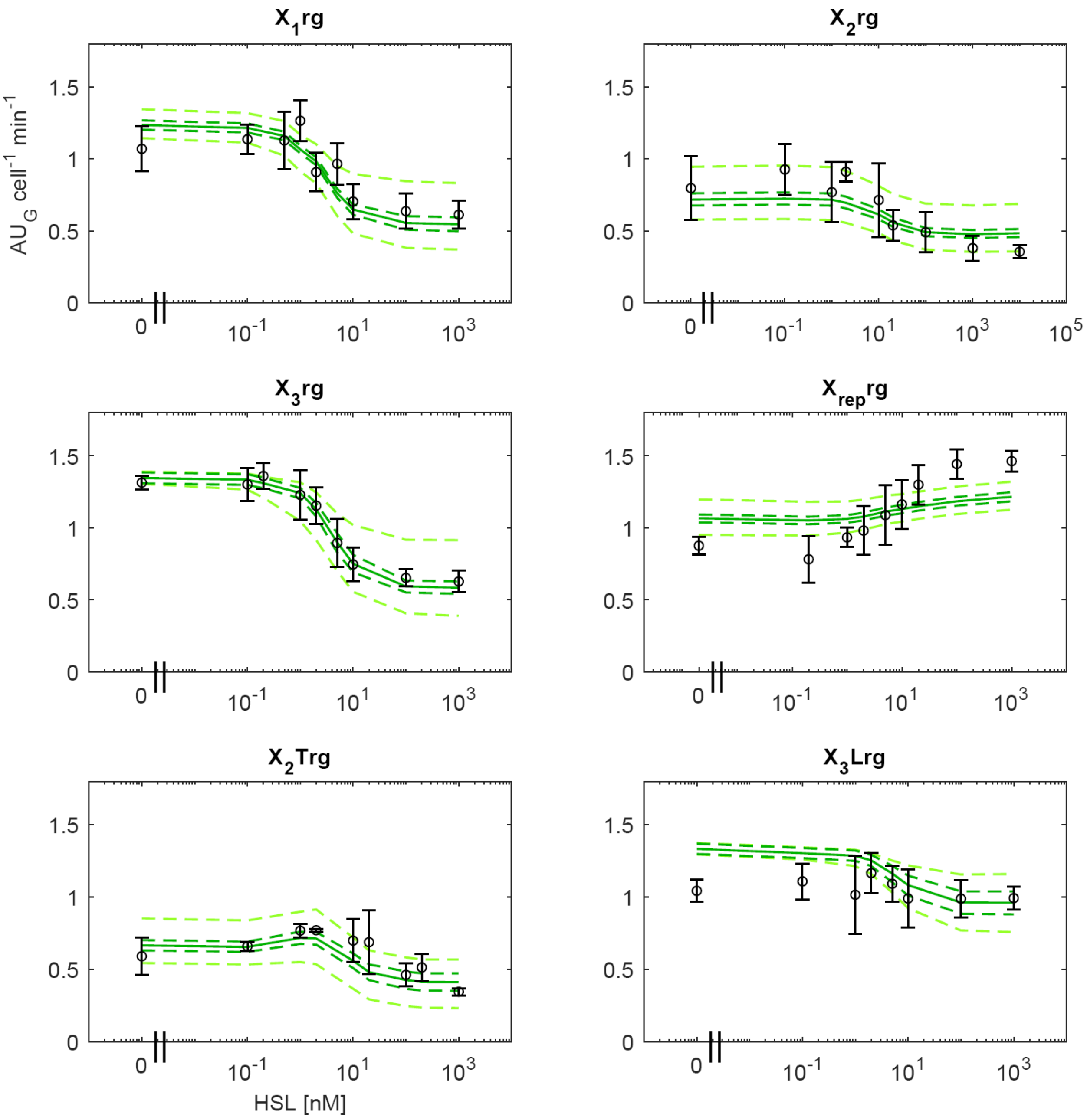

Figure S19. Results of fitting using all the available data (training and test set) using NBM and BM: RFP data. Fitting of the measured HSL-dependent RFP output in all the circuits with Monitor cassette. Circles represent the average measured value and error bars represent the 95% confidence intervals of the mean. Solid lines represent the median predicted output of the NBM (magenta) and BM (blue) calculated via Monte Carlo simulations for each HSL concentration tested.

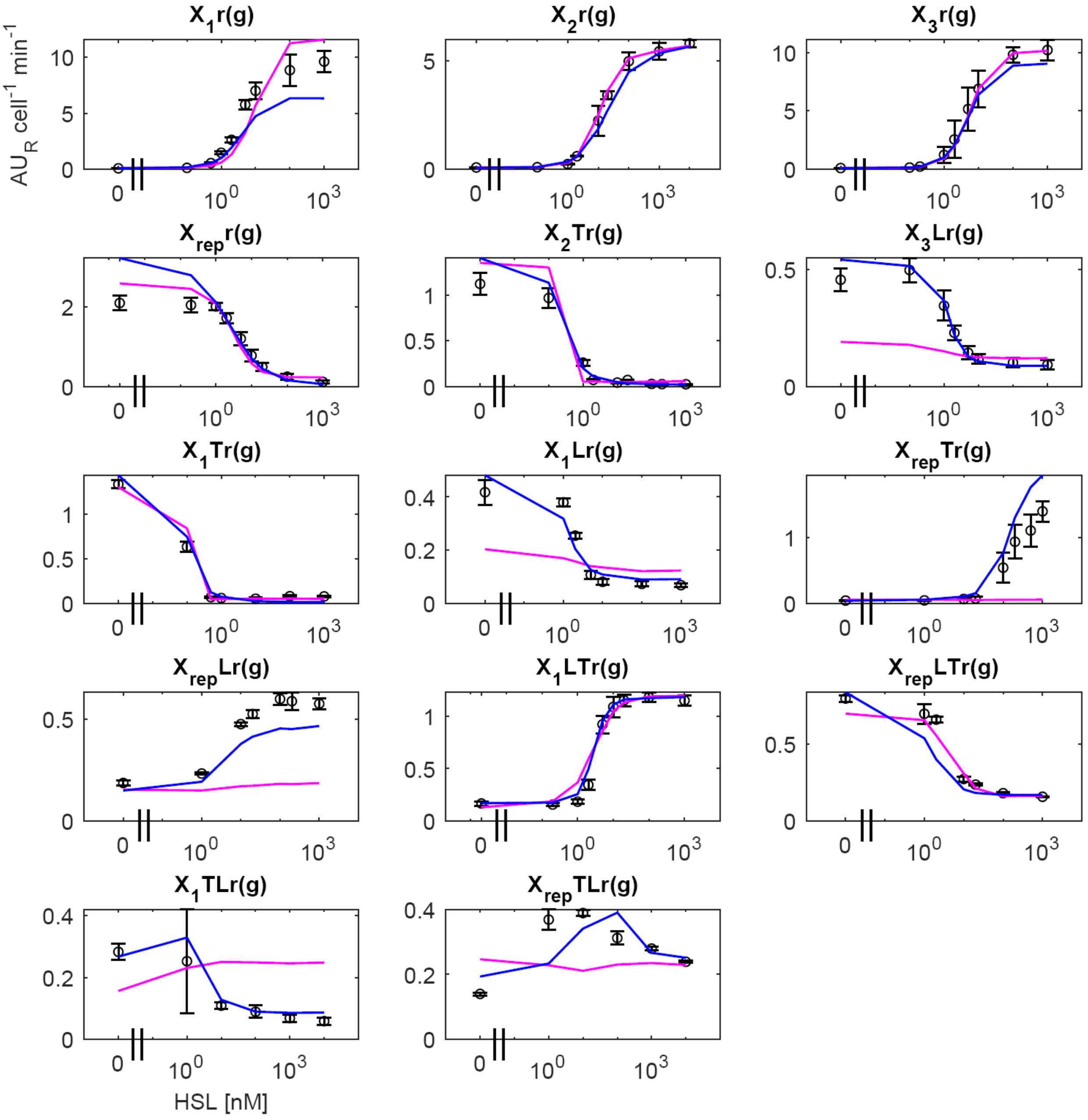

Figure S20. Results of fitting using all the available data (training and test set) using BM: GFP data. Fitting of the measured HSL-dependent GFP output in all the circuits with Monitor cassette. Circles represent the average measured value and error bars represent the 95% confidence intervals of the mean. Solid lines represent the median predicted output of the BM calculated via Monte Carlo simulations for each HSL concentration tested.

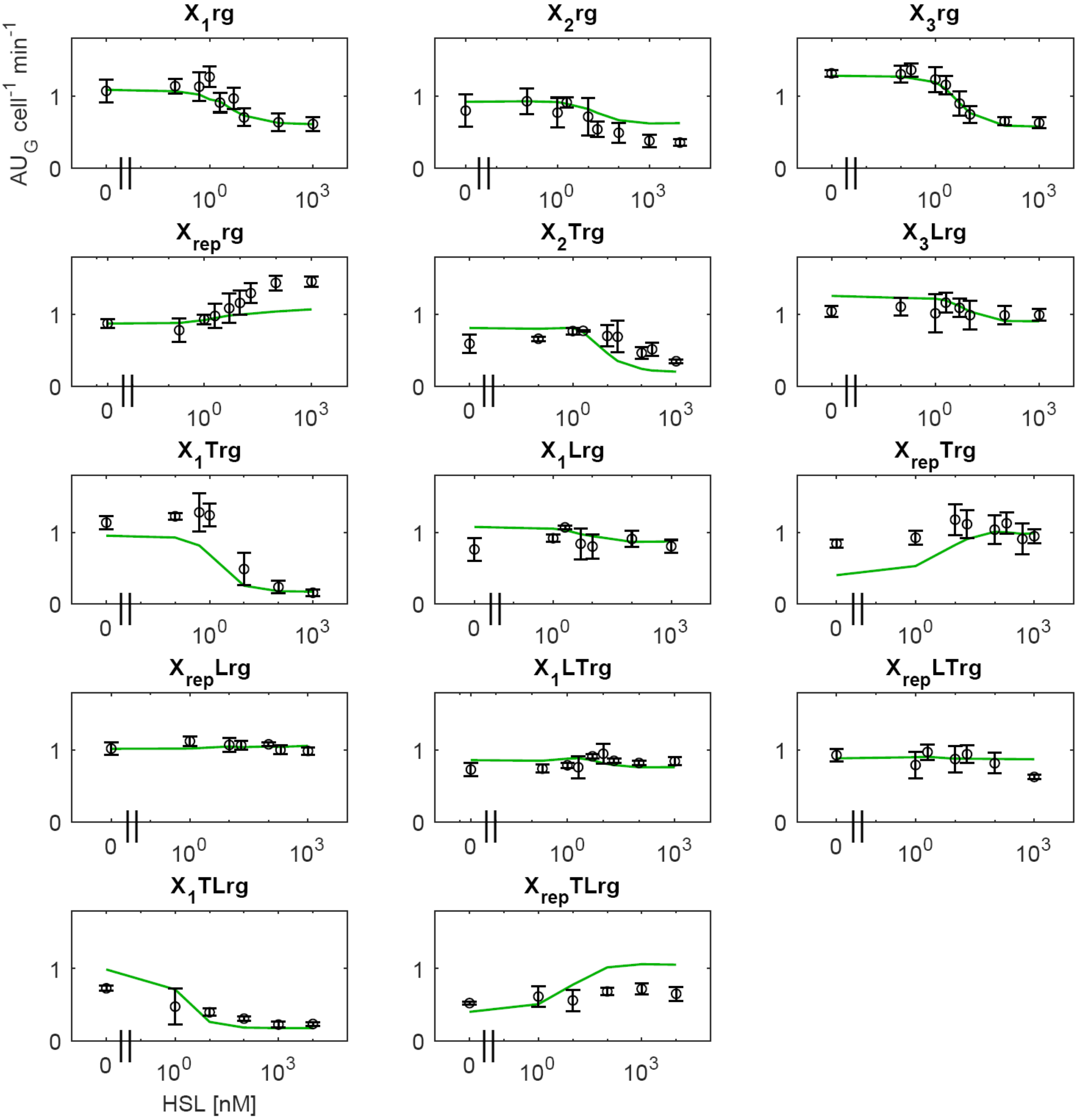

Figure S21. OD_{600}, raw GFP and raw RFP values measured in culture, supernatant and pellet of three strains. TOP10 (black; non-fluorescent), X_2Tr (red; expressing RFP) and $X_{rep}Tr$ (blue; containing RFP, not expressed) were inoculated as described in the Methods section; the 100-fold dilution was carried out in a final volume of 6 ml in 50-ml tubes and the cultures were incubated in the same conditions as before until they reached an OD_{600} of about 0.05. Then, they were sampled every 2 hours. At each sampling time, absorbance, GFP and RFP were measured for 200 µl of culture; then, 1 ml was withdrawn, transferred into a 1.5-ml tube and centrifuged (13,000 rpm, 2 min). Absorbance, GFP and RFP were measured in the supernatant (200 µl). Finally, supernatant was discarded, pellet was resuspended with 1 ml of fresh medium and absorbance, GFP and RFP were measured (200 µl). Green data points and dotted line represent the raw GFP of the medium without cells. All the measurements were carried out with the Infinite F200 reader (Tecan), as described in the Methods section. The reported data show that the raw GFP autofluorescence is due to the supernatant (see raw GFP in culture and supernatant), not to the cell pellet (see raw GFP in the resuspended pellet), although it increases during cell growth. On the other hand, red fluorescence is due to cell RFP expressing cells in the pellet.

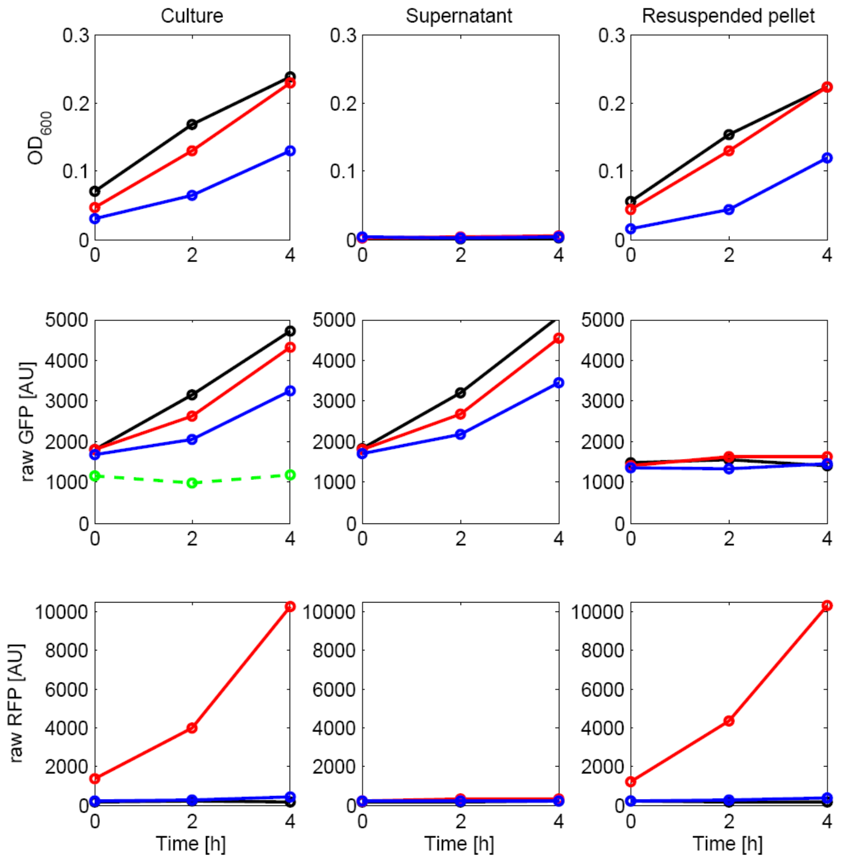

Figure S22. Raw GFP autofluorescence depends on OD_{600} and also cell growth rate. A) OD_{600} vs raw GFP characteristic of two strains showing fast (relative to the whole circuit collection) growth (X_2Tr and X_{rep}r; black and red diamonds, respectively) and two strains showing slow growth (X_{rep}Tr and X_{rep}TLr; magenta and blue squares, respectively) without HSL. They are reported, as single biological replicate, as an example to highlight distinct characteristics, dependently on growth rate. Curves like the ones shown here were fitted (with exponential regression), estimating m and q parameters (see Methods section) to obtain the autofluorescence background of the circuits. B-C) The m and q parameters for all the circuits in all the conditions are plotted against growth rate values. The m parameter (B) shows a significant growth rate-dependent trend, as expected from Panel A, while the growth rate-dependent trend of the q parameter (C) is not statistically significant (confidence intervals of the slope include zero).

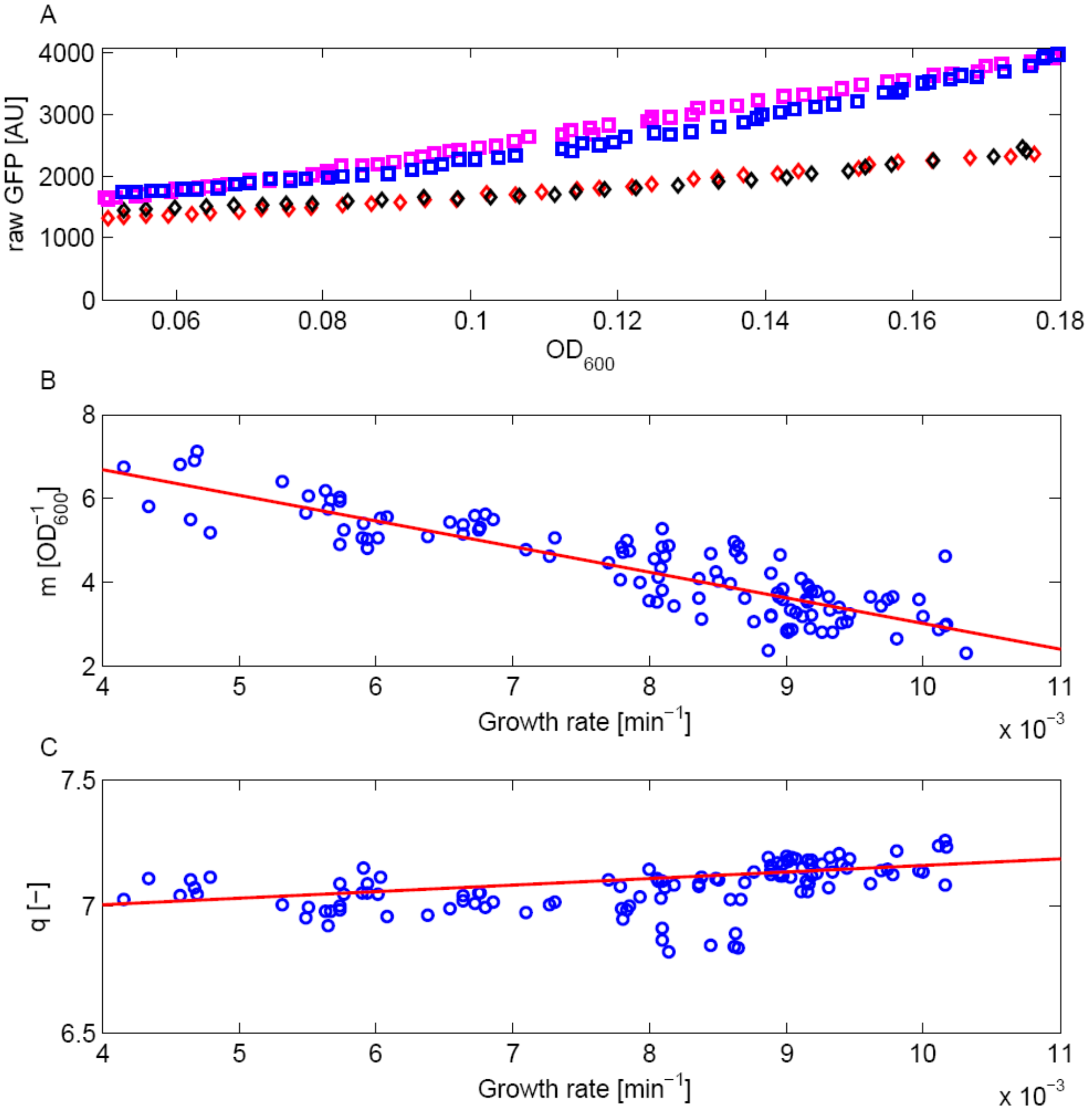

Supplementary Tables

Table S1. Parts used in this study.

Name	BioBrick code
P_{lux}	BBa_R0062
P_{luxRep}	BBa_J107100
P_R	BBa_R0051
P_{LlacO1}	BBa_R0011
P_{LtetO1}	BBa_R0040
Constitutive promoter of the Monitor cassette	BBa_J23100
Reference constitutive promoter	BBa_J23101
Strong RBS	BBa_B0030
Weak RBS	BBa_B0031
Medium RBS used in the Monitor cassette	BBa_B0032
Weak RBS used to decrease tetR translation	BBa_B0033
Strong RBS	BBa_B0034
Double transcriptional terminator	BBa_B0015
Synthetic transcriptional terminator	BBa_B1006
LuxR coding sequence	BBa_C0062
LacI coding sequence	BBa_C0012
TetR coding sequence	BBa_C0040
mRFP1 coding sequence	BBa_E1010
GFPmut3b coding sequence	BBa_E0040

Table S2. Statistics on circuits bearing the burden monitor.

**Circuit**	**Growth rate vs GFP**[*]	**RFP vs GFP**
X_1rg	<0[‡]	**-0.68**
X_2rg	0.02	**-0.8**
X_3rg	<0	**-0.9**
X_{rep}rg	<0	**-0.8**
X_2Trg	**0.41**[*]	>0
X_3Lrg	<0	>0
X_1Lrg	<0	>0
X_1Trg	**0.72**	>0
X_{rep}Lrg	<0	-0.23
X_{rep}Trg	<0	>0
X_1LTrg	<0	>0
X_1TLrg	**0.84**	>0
X_{rep}LTrg	<0	>0
X_{rep}TLrg	<0	>0

[*]Statistically significant correlation coefficients are reported in bold.

[‡]Correlation coefficients with opposite sign than expected are reported as <0 and >0 for growth rate vs GFP and RFP vs GFP, respectively.
